# Prodigiosin inhibits the proliferation of glioblastoma by regulating the KIAA1524/PP2A signaling pathway

**DOI:** 10.1038/s41598-022-23186-w

**Published:** 2022-11-02

**Authors:** Wenguo Zhao, Dezheng Gao, Liping Ning, Yousheng Jiang, Zhao Li, Bin Huang, Anjing Chen, Chuanwei Wang, Yuguang Liu

**Affiliations:** 1 Department of Neurosurgery, Qilu Hospital of Shandong University, Jinan, Shandong Province, China; 2 Department of Neurosurgery, Qilu Hospital, Cheeloo College of Medicine and Institute of Brain and Brain-Inspired Science, Jinan, Shandong Province, China; 3 Shandong Key Laboratory of Brain Function Remodeling, Jinan, Shandong Province, China; 4 Department of Rehabilitation Medicine, Shandong Provincial Hospital Affiliated to Shandong First Medical University, Jinan, Shandong Province, China

**Keywords:** Cancer, Drug discovery

## Abstract

Prodigiosin (PG), a member of a family of natural red pigments produced by a variety of bacteria, was first discovered in *Serratia marcescens*. PG has been reported to have an apoptosis-inducing effect in many cancers, such as lymphoma, colon cancer and nasopharyngeal carcinoma. For this study, we used three glioblastoma (GBM) cell lines (LN229, U251 and A172) to explore the effect of prodigiosin on GBM cells. A CCK8 assay was used to evaluate cell viability. We determinedthe cell cycle distribution by flow cytometry and measured proliferation by an EdU incorporation assay. The expression of different molecules was investigated by western blotting and RT-PCR. We further confirmed our results by plasmid transfection and lentiviral transduction. The LN229 xenograft model was used to study the effect of prodigiosin in vivo. We confirmed that prodigiosin played an anticancer role in several GBM cell lines through the KIAA1524/PP2A/Akt signalling pathway. Prodigiosin inhibited the protein expression of KIAA1524 by suppressing its transcription, which led to activation of PP2A. Afterward, PP2A inhibited the phosphorylation of Akt, thereby inducing increased expression of p53/p21. Furthermore, it was verified that prodigiosin inhibited the KIAA1524/PP2A/Akt axis in vivo in the LN229 xenograft model. These data improve the understanding of the anticancer effects of prodigiosin and further highlight the potential of prodigiosin for the development of anti-glioma drugs.

## Introduction

Glioblastoma (GBM) is one of the most malignant tumors in the human central nervous system (CNS). In 2004, the European Organization for Research and Treatment of Cancer (EORTC) and the National Cancer Institute of Canada (NCIC) Clinical Trials Group reported that surgery combined with temozolomide and radiotherapy can effectively increase the median survival time of patients^1^. Another study published by Stupp R and colleagues (2009) found that overall survival time can be significantly increased by temozolomide and radiotherapy compared with radiotherapy alone. As the primary chemotherapeutic agent for malignant gliomas, temozolomide has benefited many patients with these tumors^2^. However, the median survival time of patients is only 15 months despite treatment with adjuvant radiotherapy (RT) combined with temozolomide (TMZ) after surgical resection^3^. In addition to medicines, tumor-treating fields (TTFs) are promising treatment for GBM^4^. A clinical trial verified that treatment with alternating electric fields along with TMZ can increase the median survival time to 24.9 months^5^. In addition to exerting antimitotic effects, TTFs have been reported to affect a number of biological processes such as autophagy, immunological responses and cell migration^6^.

In Addition to current therapy, immunotherapy and targeted therapy are also promising fields for further study^7,8^. In regard to medicines, bevacizumab was once believed to be a promising therapeutic agent and was approved by the US Food and Drug Administration (FDA) in 2009. By targeting vascular endothelial growth factor (VEGF), bevacizumab can increase the median progression-free survival time to 10.6 months^9^. Currently, research on the development of new drugs or new uses of old medicines for the treatment of glioblastoma is still needed. In recent years, prodigiosin (PG) has attracted the attention of researchers as a potential antitumor drug for cancer therapy. PG has been reported to have an anticancer effect on many tumors, such as colon cancer^10^ and nasopharyngeal carcinoma^11^. Several mechanisms involved in the anticancer effects of PG have been verified, including the induction of intracellular acidification and DNA cleavage, inhibition of cell cycle progression and modulation of MAPK and Akt^12^. Regarding signaling pathways, most studies have focused on the downstream MAPK and Akt pathways, although it remains unknown how PG regulates the activity of Akt^13^.

Over the past decades, overexpression of the KIAA1524 gene, which encodes cancerous inhibitor of protein phosphatase 2A (CIP2A) has been reported in various malignancies, including gastric, bladder, ovarian, hepatocellular, colon, non–small cell lung carcinoma (NSCLC) and chronic myelogenous leukemia^14^. Some of these studies established overexpression of this molecule as a prognostic marker^15,16^. KIAA1524 is the key inhibitor of PP2A, a protein phosphatase that can regulate MYC and Akt phosphorylation and therefore affect tumor growth^17,18^. One study reported that CHK1 can activate the pSTAT3-CIP2A oncogenic circuit in GBM, which prompted us to hypothesis that the growth of GBM may be inhibited by targeting KIAA1524^19^.

In this study, we found that PG upregulated PP2A activity by suppressing KIAA1524 transcription, thereby promoting G_1_ arrest following downregulation of Akt phosphorylation. These findings contribute to our understanding of the anticancer effects of PG and further highlight the potential of this agent in antiglioma drug development.

## Results

### PG inhibited GBM cell proliferation and induced cell cycle arrest

**Figure 1:**
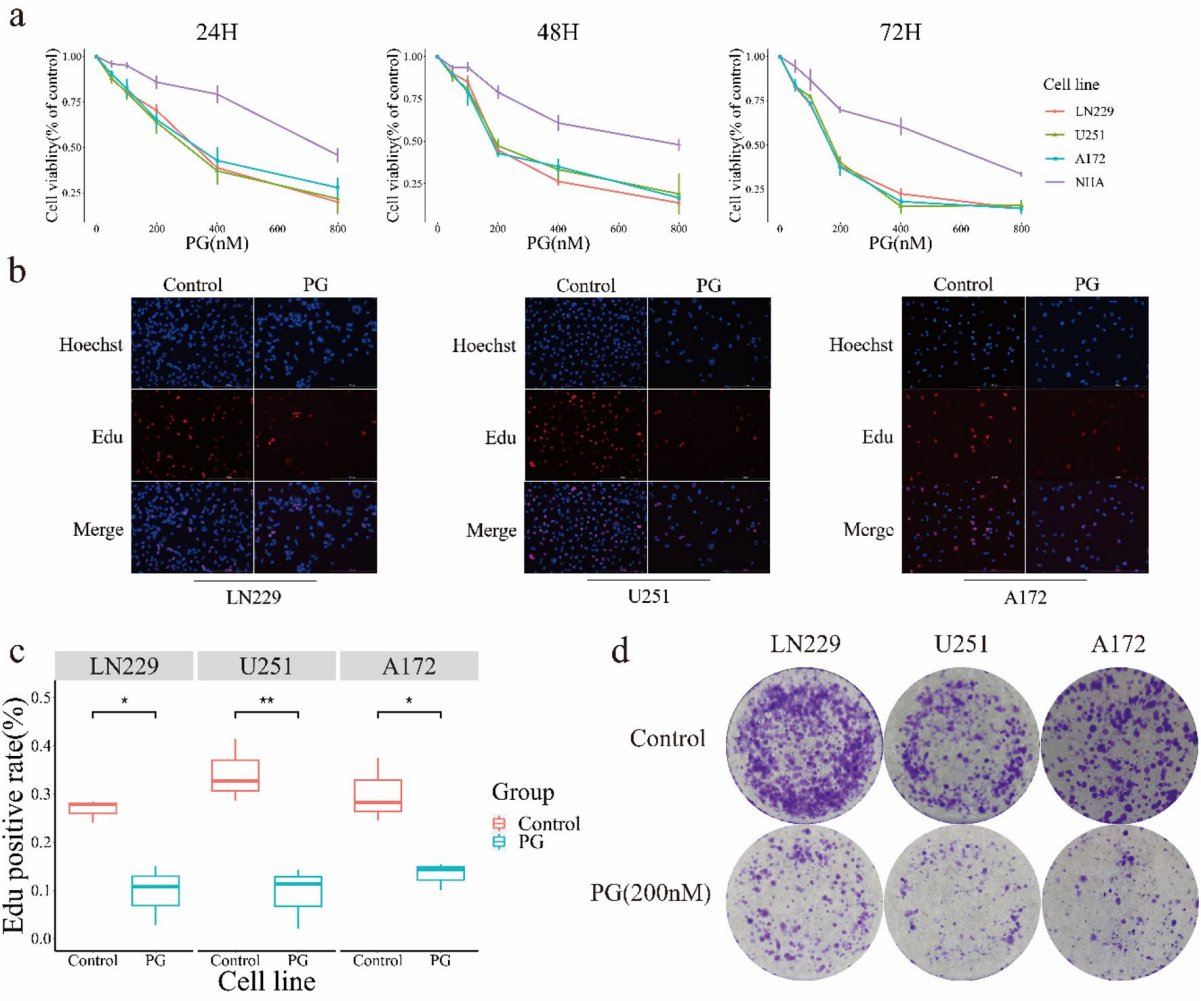
PG inhibited GBM cell proliferation and induced cell cycle arrest (**a**) PG inhibited the viability of GBM cells in a dose-dependent manner: cell viability was detected by the CCK8 assay at a specific time points after treatment with different doses of prodigiosin. (**b**) EdU immunofluorescence staining of LN229, U251 and A172 cells treated with or without PG 48 h. Cells were stained with Apollo 567 (red). Hoeschst (blue) was used to stain nuclei. (**c**) Quantification of EdU + cells (EdU positive/Hoechst positive × 100%) in all three GBM cell lines (*p < 0.05, **p < 0.01). (**d**) Images of colony formation assays for LN229, U251 and A172 cells after treating with PG (200 nM) for 2 weeks.

To determine the effects of PG on GBM cell lines (LN229, U251 and A172) and normal human astrocytes (NHAs), all cells were first treated with different concentrations of PG, and a CCK8 assay was then used to assess cell viability after 24, 48 and 72 h. As shown in Fig. 1a, PG dose-dependently reduced cell viability. An EdU incorporation assay and colony formation assay were performed on GBM cells for further study. The EdU assay indicated that PG inhibited the proliferation of GBM cells (Fig. 1b). The percentage of EdU^+^ cells treated with PG was significantly decreased compared to that cells treated with DMSO (Fig. 1c). The colony formation assay showed that the colony numbers were decreased in all three GBM cell lines (Fig. 1d). Overall, these results showed that PG inhibited the proliferation of GBM cells.

### PG induced cell cycle arrest by inhibiting the phosphorylation of Akt

**Figure 2:**
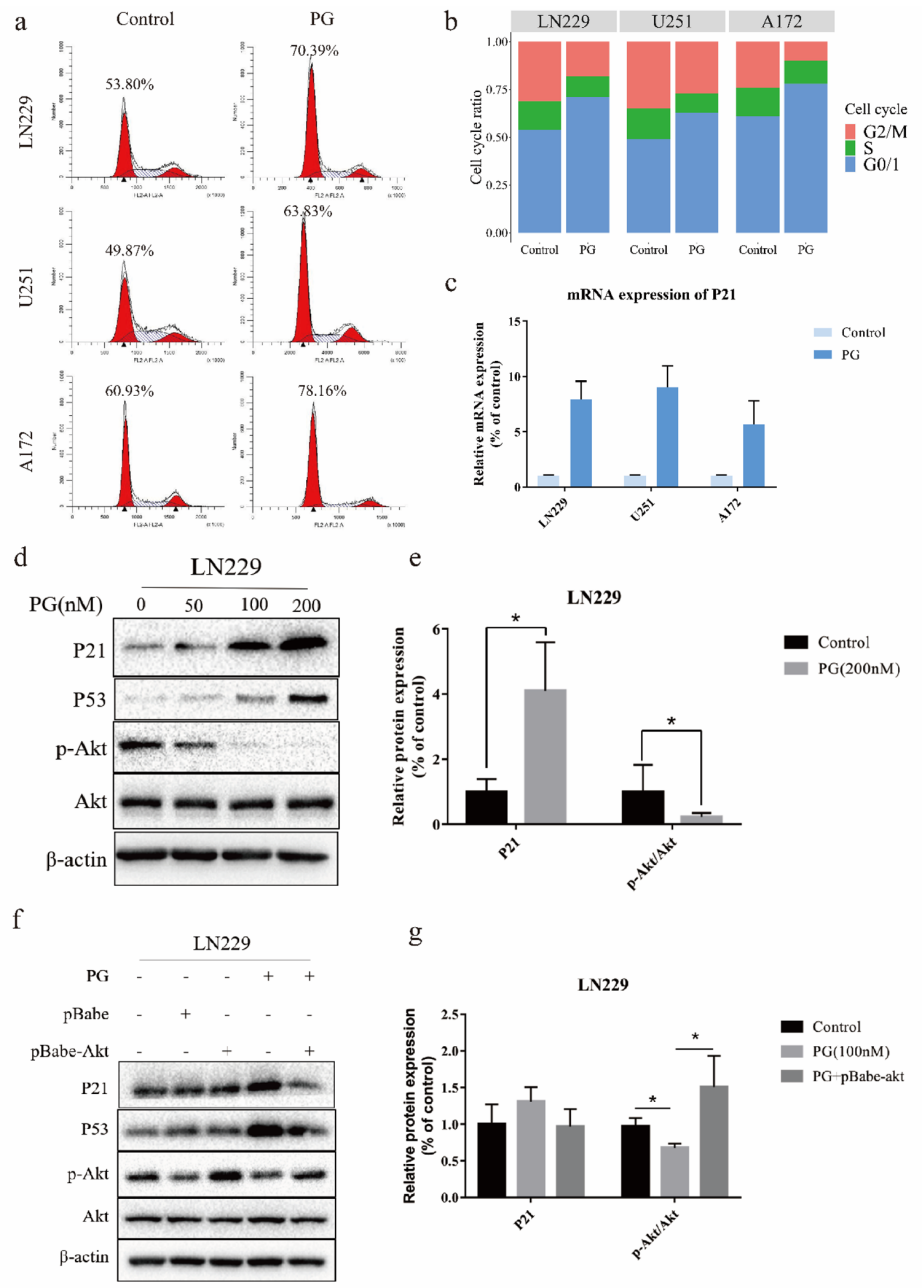
PG induced cell cycle arrest by inhibiting the phosphorylation of Akt (**a**) Cell cycle analysis of A172, LN229 and U251 cells by flow cytometry. All cells were exposed to 0 or 200 nM PG for 48 h. (**b**) Statistical data of cell cycle assay. All experiments were repeated three times. (**c**)The mRNA level of P21 was analyzed by RT-PCR. Data were expressed as the means ± SD. (**d**) The protein levels of P21, P53, p-Akt (S473), Akt and β-actin in LN229 cells treated with PG. Human β-actin was used as a reference gene. Experiments were performed three times. (**e**) Graphic representation of relative levels of protein expression in LN229 cells treated with PG. Data are expressed as the means ± SD. *p* < 0.05. (**f**) Western blotting analysis of the target proteins in LN229 cells expressing p-Akt or vector in the presence or absence of PG (100 nM) for 48 h. Experiments were performed three times. (**g**) Graphic representation of relative levels of protein expression in LN229 cells treated with PG with or without pBabe-Akt. Data are expressed as the means ± SD. *p* < 0.05.

Flow cytometry was applied to analyze the cell cycle distribution after cells were treated with PG for 48 h. Marked G_0_/G_1_ arrest was observed after treatment with 200 nM PG (Fig. 2a,b). As PG can significantly induce cell cycle arrest in GBM cells, we evaluated the expression of P21 in the three GBM cell lines by RT‒PCR. The results showed that P21 expression was greatly increased after cells were treated with PG (Fig. 2c). As it has been proven that the phosphorylation of Akt plays an important role in the regulation of the cell cycle regulatory P53/P21 pathway, we treated cells with different concentrations of PG and examined the expression levels of these proteins in all three GBM cell lines. As shown in Fig. 2d and Supplemental Fig. 1a, PG decreased the protein level of phosphorylated Akt (S473) in a dose-dependent manner while the protein level of Akt did not change in any of these GBM cell lines. The expression of P53 and P21 was also changed in a dose-dependent manner. Quantitative statistical analysis of the P21 and p-Akt/Akt protein levels shown in Fig. 2e indicated significant differences between cells with and without PG treatment. To further study whether PG performs its function through phosphorylation of Akt, we transfected LN229 cells with an Akt overexpression plasmid. Western blotting verified that PG blocked the cell cycle by inhibiting the phosphorylation of Akt and promoting the expression of P53 and P21, and this effect was reversed by increasing the level of phosphorylated Akt (Fig. 2f,g). The results of flow cytometric analysis of the cell cycle shown in Supplemental Fig. 1b also proved that the phosphorylation of Akt played an important role in the cell cycle arrest induced by PG.

### PG induced GBM cell cycle arrest by inhibiting the KIAA1524/PP2A signaling pathway

**Figure 3:**
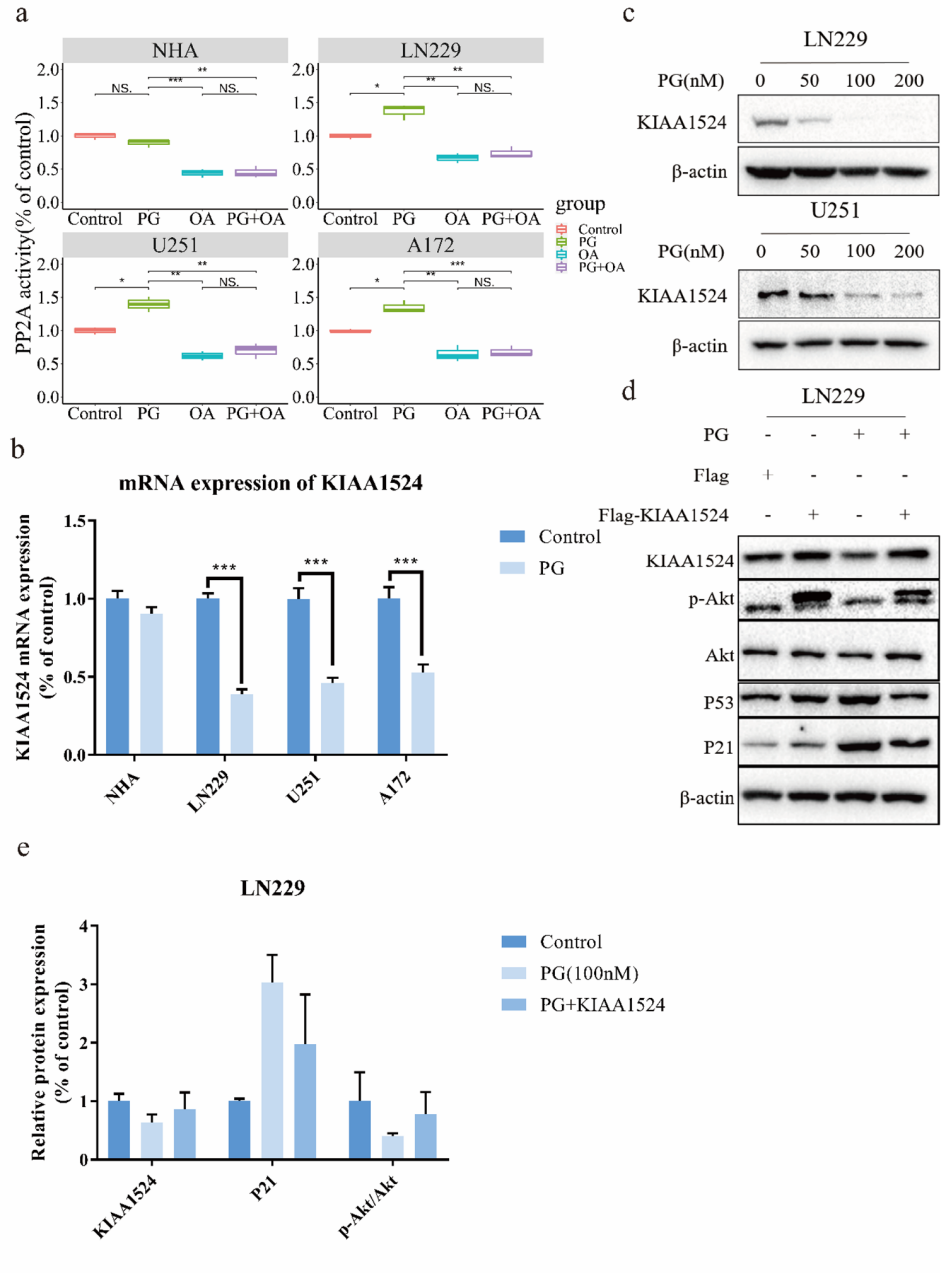
PG induced GBM cell cycle arrest by inhibiting the KIAA1524/PP2A signaling pathway (**a**) The activity of PP2A in GBM cells was increased after exposure to PG: GBM cells were treated with PG, OA or both at 100 nM for 24 h, and cell lysates were extracted to assess the activity of PP2A. (**b**) RT-PCR analysis of KIAA1524 expression in LN229, U251 and A172 cells treated with or without PG. (**c**) Western blotting analysis of KIAA1524 expression in LN229 and U251 cells treated with or without PG. (**d**) Western blotting analysis of the target proteins after ectopic expression of KIAA1524 in LN229 cells. Experiments were performed three times. (**e**) Graphic representation of relative levels of protein expression in LN229 cells treated with PG with or without KIAA1524. Data are expressed as the means ± SD. *p* < 0.05.

To ascertain how PG inhibits the phosphorylation of Akt (S473), we first explored the expression of PTEN and PHLPP, which can inhibit Akt phosphorylation. As shown in Supplemental Fig. 2a, their expression did not change significantly after cells were treated with PG. These results indicated that PG may not exert its anticancer effects by affecting the expression of PTEN or PHLPP. We then evaluted the activity of PP2A in all GBM cell lines and NHAs. As shown in Fig. 3a, after exposure to PG (200 nM) for 24 h, the activity of PP2A was found to obviously increased in all GBM cell lines, and this increase was reversed by treatment with okadaic acid (OA; an inhibitor of PP2A). However, the activity of PP2A did not change in NHAs after treatment with PG. Therefore, we assumed that PG exerted its effects by promoting the activity of PP2A. It has been reported that KIAA1524 (CIP2A, an oncogenic inhibitor of PP2A) is an endogenous inhibitor of PP2A. KIAA1524 mRNA expression was examined by RT‒PCR to further confirm this assumption. The KIAA1524 mRNA level was significantly decreased in all three GBM cell lines after treatment with PG for 48 h. However, the mRNA expression of KIAA1524 in NHAs treated with PG differed only slightly from that in control cells (Fig. 3b). Western blotting was performed to evalute the protein levels of KIAA1524 after cells were exposed to different concentrations of PG (Fig. 3c and Supplemental Fig. 2b). Collectively, theresults supported the hypothesis that PG can inhibit the expression of KIAA1524. To further verify the idea that the level of KIAA1524 plays an important role in the anticancer effect of PG, the expression of KIAA1524 in the LN229 cell line was upregulated bylentiviral transduction. The change in protein expression induced by PG was reversed by exogenous expression of KIAA1524 (Fig. 3d,e).

### Bioinformatics analysis of KIAA1524

**Figure 4:**
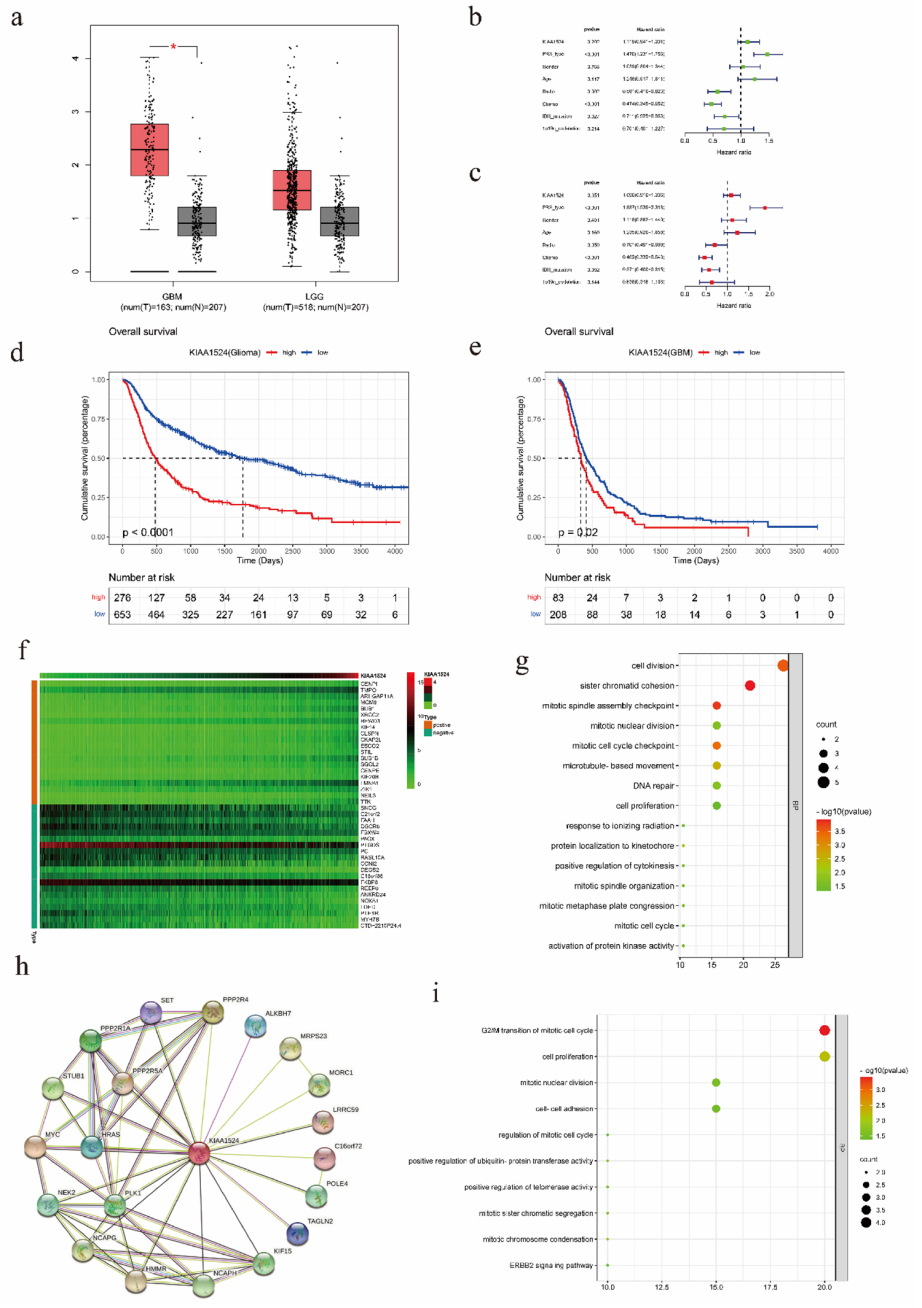
Bioinformatics analysis of KIAA1524 (**a**) The mRNA level of KIAA1524 was analyzed by Gene Expression Profiling Interactive Analysis (GEPIA). (**b**, **c**) Univariate and multivariate Cox analyses of KIAA1524 expression and other clinical pathological factors. (**d**, **e**) Survival curve of differential KIAA1524 expression. (**f**) Heatmap of top20 KIAA1524 associated genes in glioma. (**g**) GO analysis of top20 KIAA1524 positively related genes in glioma. (**h**) Protein interaction of KIAA1524. (**i**) GO analysis of KIAA1524 interacting molecules.

As KIAA1524 may play an important role in the proliferation inhibitory function of PG, we analyzed KIAA1524 expression in gliomas with Gene Expression Profiling Interactive Analysis (GEPIA). KIAA1524 mRNA expression was high in LGG and GBM (Fig. 4a). We then explored the function of KIAA1524 using the CGGA RNA-seq dataset^20^. Univariate and multivariate analyses (Fig. 4b,c) showed that KIAA1524 expression (*p* < 0.001), alone or in combination with PRS-type (*p* < 0.001), grade (*p* < 0.001), patient age (*p* < 0.05), IDH mutation status (*p* < 0.01) and 1p19q codeletion status (*p* < 0.001), was significantly associated with overall survival. Survival analysis showed that in both the glioma and GBM groups, patients with higher KIAA1524 expression had shorter OS time (Fig. 4d,e). A heatmap (Fig. 4f) of the top 20 genes whose expression was positively and negatively associated with KIAA1524 expression was generated. GO functional enrichment analysis of the molecules positively related to KIAA1524 showed association with cell cycle and cell proliferation (Fig. 4g). The protein interactions of KIAA1524 were plotted with the STRING database (Fig. 4h). We also analyzed the biological processes of these proteins and found a relationship with the cell cycle and cell proliferation. Additionally, GO functional enrichment analysis (Fig. 4i) revealed that KIAA1524 was related to cell cycle regulation. The above results suggest that KIAA1524 plays an important role in glioma progression.

### Prodigiosin suppresses KIAA1524 transcription by disrupting the binding of ELK1 to the KIAA1524 promoter

**Figure 5:**
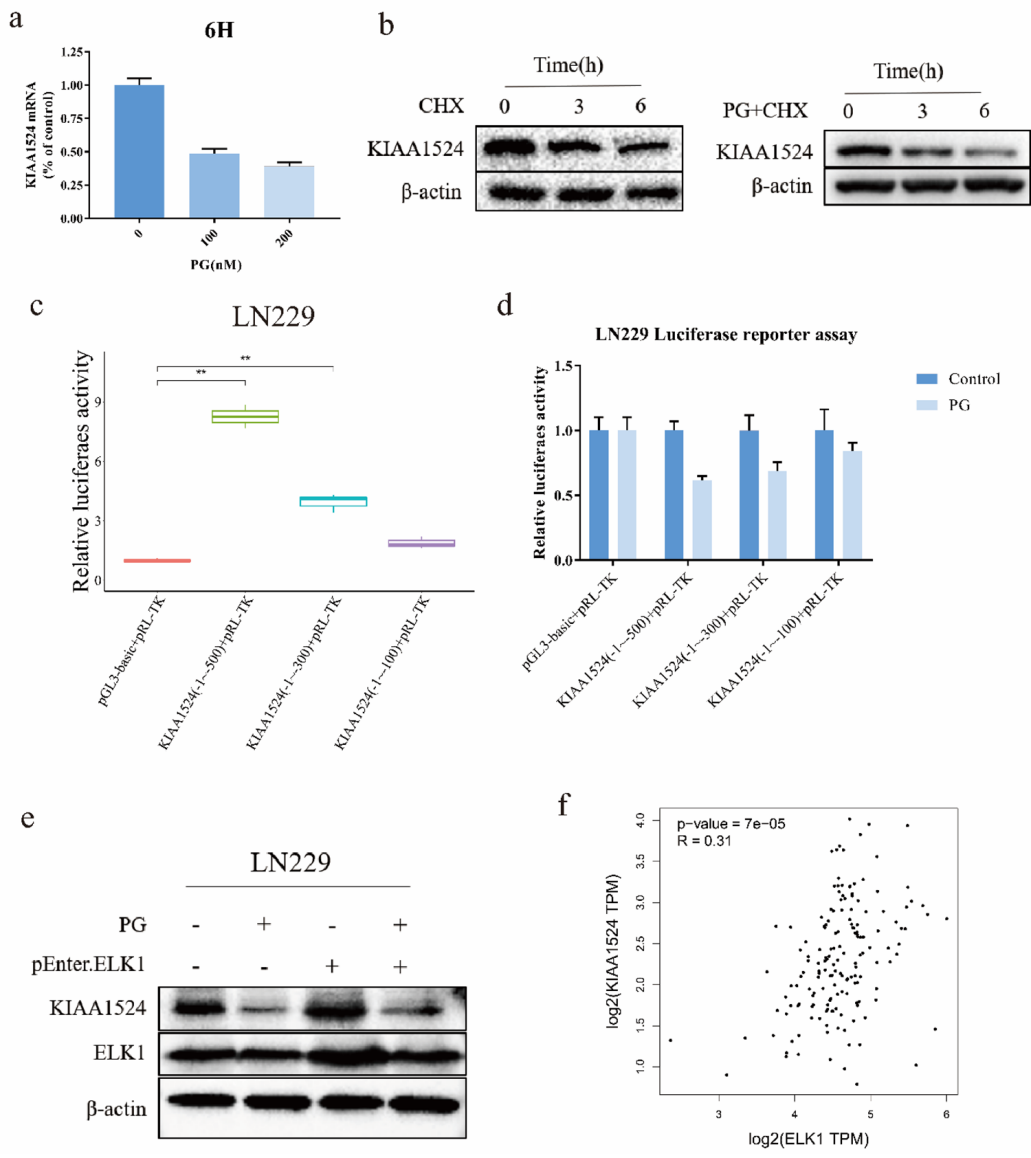
Prodigiosin suppresses KIAA1524 transcription by disrupting the binding of Elk-1 to the promoter of KIAA1524. (**a**) Quantification of KIAA1524 mRNA level in LN229 cells treated with or without PG for 6 h. (**b**) The protein level of KIAA1524 in LN229 cells treated with 100 µg/ml CHX with or without PG for 0 h, 3 h or 6 h. (**c**, **d**) Effects of PG on KIAA1524 promoter activity. LN229 cells were co-transfected with different promoter regions before being treated with PG. Cell lysates were prepared for analysis of luciferase activity. (* *p* < 0.05. ** *p* < 0.01). (**e**)Ectopic expression of ELK1 reversed the effects of PG in GBM cells: ELK1 was up-regulated by transfection. Cells were treated with or without PG at 100 nM for 24 h and analyzed by western blotting. (**f**) Bioinformatics correlation analysis between KIAA1524 expression and ELK1.

To determine how PG inhibits KIAA1524, we examined the RNA and protein expression of KIAA1524. The RT‒PCR results showed that the KIAA1524 mRNA level was significantly decreased after treatment with PG (200 nM) for 6 h (Fig. 5a). However, after protein synthesis was blocked by CHX, the protein expression of KIAA1524 did not differ greatly in cells treated with or without PG (Fig. 5b). Therefore, we presumed that PG inhibits the expression of KIAA1524 by suppressing its transcription. A luciferase reporter assay was used to study how PG regulates the transcription of KIAA1524. LN229 cells were transfected with plasmids containing different KIAA1524 promoter regions (− 1 ~  − 100 bp, − 1 ~  − 300 bp, − 1 ~  − 500 bp), and the activity of each promoter region except for − 0 ~  − 100 bp region was found to be significantly reduced by PG (Fig. 5c,d). As it has been reported that ETS transcription factor ELK1 can bind to the promoter of KIAA1524 at bp − 127 to − 137 to initiate KIAA1524 transcription^21,22^, we assumed that PG might interfere with the binding of ELK1. LN229 cells were transfected with an ELK1 overexpression plasmid to test this hypothesis. As shown in Fig. 5e, ectopic expression of ELK1 restored KIAA1524 expression, which was inhibited by PG. The expression of ELK1 and KIAA1524 in GBM was also analyzed with GEPIA (Fig. 5f). The expression of KIAA1524 was correlated with that of ELK1 in GBM tissues. Collectively, these studies proved the hypothesis that PG can inhibit the transcription of KIAA1524 by affecting the binding of ELK1 to the promoter of KIAA1524.

### Antitumor effects of PG in the LN229 xenograft model

**Figure 6:**
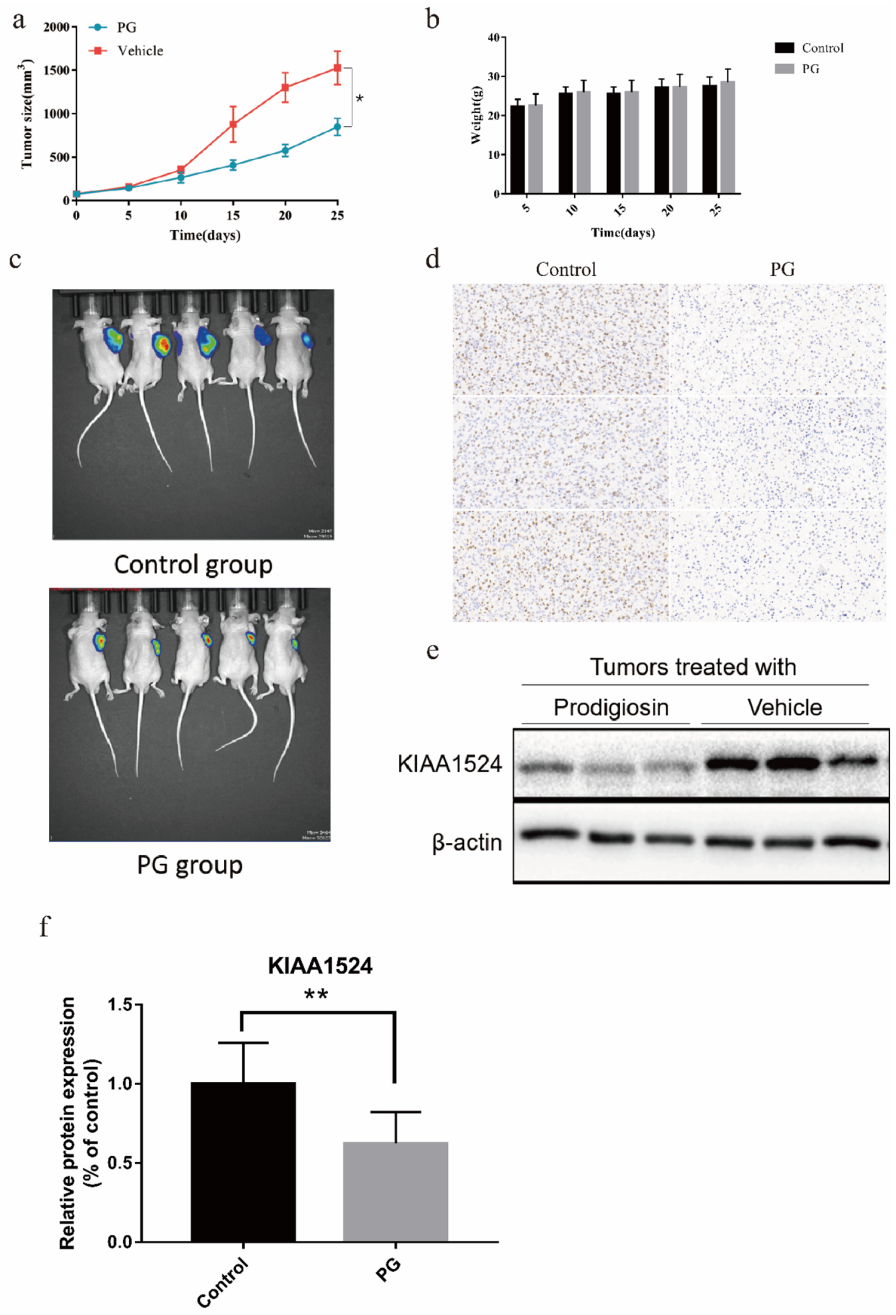
Antitumor effect of PG in the LN229 xenograft model. (**a**) The growth curves of LN229 xenograft tumors in PG and vehicle-treated nude mice: the size of tumors was measured by Vernier calipers every five days. Tumor volume calculation formula: Length × width × width/2. Data are expressed as the means ± SEM. *p* < 0.05. (**b**) Graphic representation of body weight. Data are expressed as the means ± SD. *p* < 0.05. (**c**) Bioluminescent signals in mice were detected immediately after injecting D-luciferin potassium salt (Day 25). (**d**) Immunohistochemistry staining of Ki67 in the tumor sections from control or PG group.(**e**) The protein levels of KIAA1524 and β-actin in LN229 xenograft tumors were measured by western blotting. (**f**) Graphic representation of relative levels of protein expression in tumors. Data are expressed as the means ± SD. *p* < 0.05.

To demonstrate the clinical feasibility of PG, we evaluated the effects of PG in vivo using a mouse xenograft model established with the LN229 cell line. PG (5 mg/kg) or DMSO (vehicle, used as a control) was intraperitoneally injected into tumor-bearing mice once every three days. After 25 days of treatment, the tumor size in the mice receiving PG was significantly smaller than that of the mice receiving vehicle (Fig. 6a), while the body weight of mice the two groups was not significantly different. We also used PerkinElmer IVIS Spectrum to evaluate tumor size (Fig. 6c). The immunohistochemical staining results demonstrated that the expressionof the cell proliferation marker, ki67, was decreased in PG-treated tumors compared with control tumors. (Fig. 6d). Next, we harvested xenografts for western blotting. PG treatment decreased the protein level of KIAA1524 in the in vivo tumor samples (Fig. 6e,f). In conclusion, ddownregulation of KIAA1524 by PG can inhibit the development of tumors.

## Discussion

Glioblastoma (GBM) is the most common malignant primary brain tumor in adults. The current treatment strategy for GBM is radiotherapy combined with temozolomide (TMZ) after surgical resection. Temozolomide, which was initially synthesized in the 1980s as a water-soluble imidazotetrazinone^23^, causes mismatched base pairing that eventually leads to apoptosis^24^. Although TMZ improves overall survival, a large number of patients experience recurrence due to resistance to TMZ^25^. In addition to temozolomide, carmustine implants, bevacizumab, and TTF therapy have been approved by the US FDA for the treatment of GBM^26^. All these methods have been proved to benefit GBM patients in clinical trials.

Currently, difficulties in treating GBM with drugs include drug resistance, poor drug pharmacokinetic properties, tumor heterogeneity, etc. More effective ways are still needed for the treatment of GBM. Prodiginines, a family of heterocyclic tripyrroles^27^, are natural red pigments produced by a variety of bacteria. As natural secondary metabolites, the class of linear tripyrroles includes prodigiosin, which was first discovered in *Serratia marcescens*, and undecylprodigiosin^28^. These molecules have attracted much attention due to their antimicrobial, antifungal, immunosuppressive and anticancer activities. Montaner’s study (2000) showed that PG induces apoptosis in several hematopoietic cancer cell lines (Jurkat, NSO, HL-60 and Ramos) but not in nonmalignant cells (NIH-3T3 and MDCK)^29^. The same phenomenon was observed in human colon adenocarcinoma cells (DLD-1 and SW-620) and nonmalignant cells (NRK and Swiss-3T3)^30^ A study performed on MCF-7 and HDF cells showed that the anticancer ability of PG was independent of the MDR1, BCRP, or MRP transporter. This prompted us to hypothesize that PG may be a potential treatment to combat malignant cancer cells that overexpress multidrug resistance transporters. PG and its analog obatoclax were tested as new anticancer drugs in clinical trials^31^. Zahra Arshadi reported that PG can function either as a radiosensitizer or a radioprotective agent depending on its dose^32^. Our early experiments also showed that PG can exert anticancer effects on human GBM cells. Therefore, we believe that the study of PG may be conducive to the treatment of GBM. In our study, we revealed that the anti-GBM effect of PG occurs through targeting the transcription of KIAA1524.

Although PG’s antitumor effect and functions, such as inducing DNA cleavage, have already been studied in several researches^33^, it remains unclear how PG performs its function in GBM cells. In our study, we explored the function of PG both in vitro and in vivo. The function of PG was verified in vivo in a subcutaneous xenograft model. In vitro, we performed GBM cell viability assays to explore the function of PG. The results showed that PG can inhibit cell proliferation in a concentration-dependent manner. The following experiments, immunofluorescence staining and a colony formation assay, further proved the cell proliferation inhibitory function of PG. The decrease in the number of colonies formed by cells treated with PG proved the proliferation-inhibiting ability of PG. The number of EdU^+^ cells is an indicator of DNA synthesis, and the reduction in the number of EdU^+^ cells among PG treated cells indicated a decrease in cell proliferation via downregulation of DNA synthesis. As cell cycle arrest can reduce DNA synthesis in cells and is a common cause of proliferation inhibition, and PG has been reported to induce cell cycle arrest, we performed flow cytometry for further study. Flow cytometric analysis confirmed that PG blocked the cell cycle. It is worth noting that although there have been multiple studies confirming that PG affects the cell cycle, all studies to date have focused on events downstream of Akt, such as regulation the P21 pathway in a P53-dependent or P53-independent manner^34^. In human lung adenocarcinoma cells, PG also regulates P27 stabilization via phosphorylation of Akt^12^. To our knowledge, it is still unknown how PG regulates Akt. It was verified that the Akt signaling pathway is abnormally activated in GBM cells, which leads to their malignant proliferation. This means that the abnormal phosphorylation of Akt may be a promising target to inhibit the abnormal proliferation of GBM cells.

**Figure 7:**
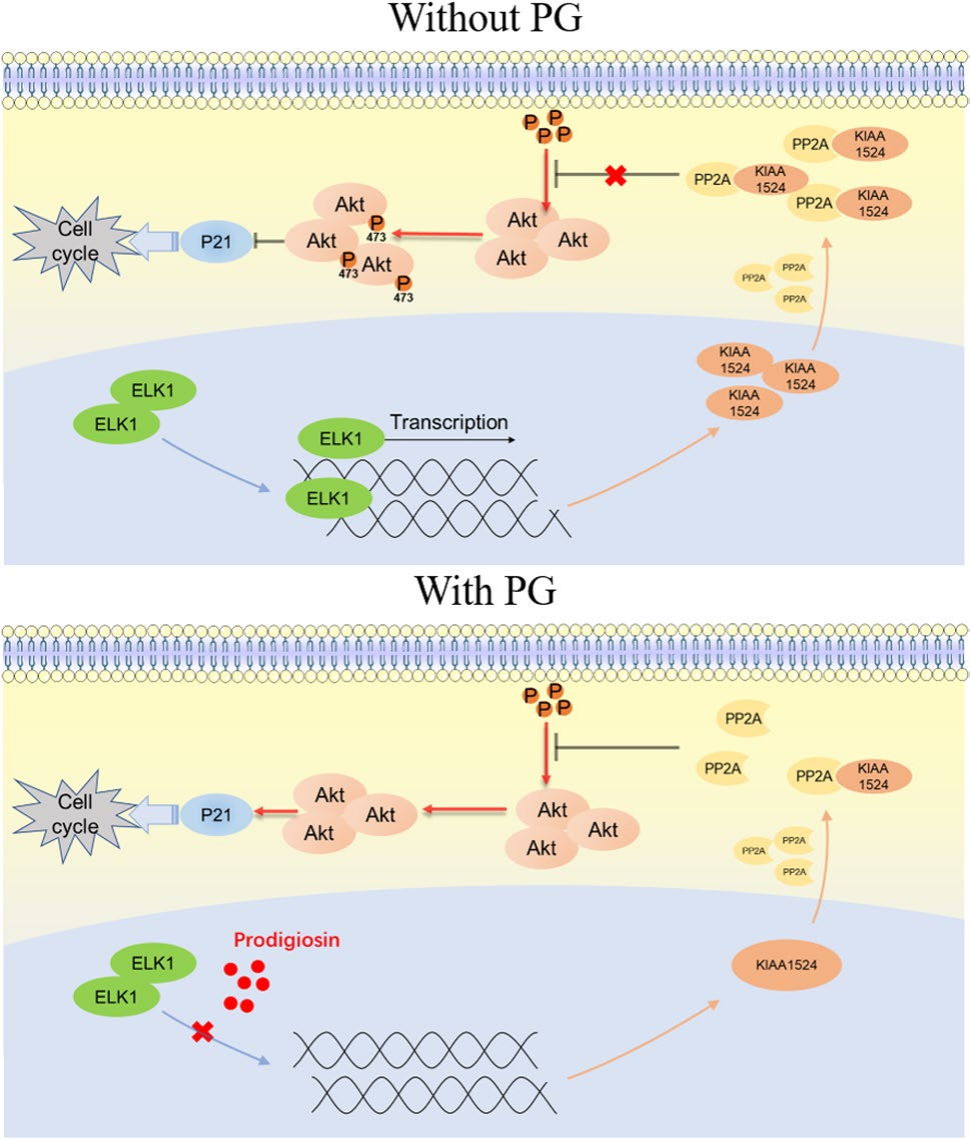
summarized signal pathway diagram of PG.

An important serine/threonine phosphatase, PP2A can regulate the cell cycle by controlling the G1/S transition. Its central role as a regulator of the cell cycle revealed its function as a tumor regulator^35^. Through a PP2A activity assay, we found that PG promoted the activity of PP2A in GBM cells, which led to dephosphorylation of Akt. In humans, KIAA1524 can directly interact with and inhibit the activity of PP2A^36,37^. This mechanism by which upregulation of KIAA1524 decreases the level of PP2A activity has been verified in several kinds of cancers, such as hepatocellular carcinoma and triple-negative breast cancer^38,39^. Western blotting proved our hypothesis that PG can inhibit the protein expression of KIAA1524. Further experiments proved that PG inhibited the expression of KIAA1524 by regulating its mRNA transcription. Our study showed that PG suppressed the expression of KIAA1524 by disrupting the interaction of ELK1 with the promotor of KIAA1524, with no effect on the protein stability of KIAA1524. To the best of our knowledge, our study is the first to reveal that the KIAA1524/PP2A/Akt axis is involved in the antiproliferative effect of PG and that PG inhibits the expression of KIAA1524 by inhibiting the transcriptional regulatory function of ELK1. A schematic of our results is shown in Fig. 7.

We also analyzed the expression of KIAA1524 in glioblastoma with a public database. We found that KIAA1524 is overexpressed in GBM and that its expression is correlated with overall survival, which means that it may be a promising target for the treatment of GBM. Enrichment analysis of genes upregulated and downregulated by PG treatment revealed that KIAA1524 may affect a variety of processes in GBM cells. In addition to inducing cell cycle arrest, it may affect cell division, DNA repair, cell‒cell adhesion and regulation of telomerase activity, and some of these functions were also reported in other studies of PG. These results indicate that PG may play a role in tumor suppression via multiple mechanisms. In our study, we also found that a high concentration of PG induced apoptosis, consistent with the findings of Cheng^40^. It is worth performing more studies on the function and mechanism of PG before it is used in clinical practice.

In summary, increased expression of KIAA1524 may be correlated with increased grade in glioma. By targeting KIAA1524, PG can suppress Akt phosphorylation and induce cell cycle arrest in GBM cells. Perhaps, PG may become a drug for GBM treatment.

## Materials and methods

### Ethics statement

All animal experiments followed the protocol approved by the Animal Research Management Committee of Shandong University. All methods are reported in accordance with ARRIVE guidelines (https://arriveguidelines.org) for the reporting of animal experiments and all methods were carried out in accordance with the relevant guidelines and regulations. Male BALB/c athymic mice were purchased from Beijing Vital River Laboratory Animal Technology Co., Ltd. All animal procedures followed the instructions of the Institutional Animal Care and Use Committee (IACUC) of Shandong University (Jinan, China).

### Cell viability assay

Human GBM cell lines (U251, A172 and LN229) and normal human astrocytes (NHAs) were obtained from ATCC (Beijing, China). U251, A172 and LN229 cells were seeded in 96-well plates at a density of 2 × 10^4^ cells/well and grown overnight. The cells were exposed to DMSO or prodigiosin at various concentrations for 48 h, and cell proliferation assays were performed using Cell Proliferation CCK8 Kit (Dojindo, Kumamoto, Japan). Experiments were performed three times independently.

### EdU immunofluorescence staining assay

The U251, A172 and LN229 cells were plated at a density of 3 × 10^4^ cells/well in 24-well plates for 24 h. Then cells were treated with or without PG (200 nM) for 48 h. The medium was discarded, and the wells were washed with sterile PBS. EdU was mixed with medium at 1:1000 dilution. and the cells for two hours. Staining was performed after the EdU incorporation assay (C103103, RiboBio; Guangzhou, China). Nuclei were stained with Hoechst. Cells were visualized by fluorescence microscopy. Experiments were performed three times independently.

### Colony Formation Assay

U251, A172 and LN229 cells were seeded in 6-well plates at a density of 1 × 10^3^ cells/well. The cells were treated with or without PG (100 nM) for two weeks. The plate were washed with PBS, and the cells were fixed with 4% paraformaldehyde for 15 min. The cells were stained for 20 min with crystal violet. Experiments were performed three times independently.

### Flow cytometry

U251, A172 and LN229 cells were seeded in 6-well plates at a density of 2 × 10^4^ cells/well and grown overnight. The cells were then exposed to DMSO or prodigiosin at a concentration of 100 nM for 48 h. The cells were digested and washed with PBS. Ice-cold ethanol (70%) was used to resuspend the cells, and the samples were refrigerated at 4 °C overnight. The cells were centrifuged and resuspended in PBS. A C6 flow cytometer was used to detect cell nuclei at an excitation wavelength of 488 nm after 15 min of PI staining. The absorbance was measured at 585 nm. Experiments were performed three times independently^27^.

### Western blot analysis

Cells were lysed in RIPA buffer with protease and phosphatase inhibitors. Equal amounts of proteins were separated by10% SDS‒PAGE, and proteins were electroblotted onto PVDF membranes. After blocking with skim milk for 1 h, membranes were cut into bands according to the molecular weight and incubated with primary antibodies (diluted in primary antibody dilution buffer) for 24–48 h at 4 °C. The membranes were then washed three times with TBST and incubated with secondary antibodies for 1 h at room temperature. Signals were visualized using a Bio-Rad ChemiDoc XRS + imaging system (Hercules, CA, USA) with ECL reagents (Merck Millipore; Billerica, MA, USA). The following primary antibodies were used: rabbit anti-KIAA1524 (#14,805, 1:1000, Cell Signaling Technology), rabbit anti-β-actin (#4970, 1:1000, Cell Signaling Technology), rabbit anti-P21 (#2947, 1:1000, Cell Signaling Technology), rabbit anti-P53 (#74,556, 1:1000, Cell Signaling Technology) rabbit anti-Elk-1 (#9182, 1:1000, Cell Signaling Technology), anti-p-Akt (S473) (#ab285140, 1:1000, Abcam) and anti-Akt (#ab8805, 1:1000, Abcam)^32^. Experiments were performed three times independently.

### Cell transfection

LN229 cells were seeded in 6-well plates at a density of 2 × 10^4^ cells/well. At approximately 60% confluence, the cells were transfected with a lentiviral vector for 48 h. KIAA1524 cDNA cloned into the LV5 vector was purchased from Biosune Biotechnology (Shanghai) Co., Ltd. The sequence of KIAA1524 can be found in supplement 3.

### PP2A activity assay

Cells were treated with PG or okadaic acid (Tocris Cookson, Ballwin, MO, USA) for 48 h. The activity of PP2A was measured with a PP2A Immunoprecipitation Phosphatase Assay Kit. Cell lysates were first prepared in a low-detergent lysis buffer according to the instructions. Next, 100 µL of Malachite Green phosphate detection solution was added, and the reaction was allowed to proceed for 15 min at room temperature. The absorbance was measured at 650 nm. Experiments were performed three times independently.

### Real-time PCR analysis

Total RNA was extracted using an RNA extraction kit (RN001-100 T) and reverse transcribed into cDNA using another kit (KR116-02) according to the kit’s instructions. The thermal cycling program used for PCR comprised three steps: denaturation at 95 °C for 10 s, annealing at 60 °C for 20 s, and elongation at 75 °C for 20 s. These three steps were repeated for 20–40 cycles to obtain numerous sequences of DNA. A Roche instrument was used for quantitative PCR analysis using the following primers: KIAA1524 (human): sense primer, 5’-TGGCAAGATTGACCTGGGATTTGGA-3’ and antisense primer, 5’-AGGAGTAATCAAACGTGGGTCCTGA-3’; β-actin (human): sense primer, 5 ‘-ATAGCACAGCCTGGA TAGCAACGTAC-3’ and antisense primer, 5’-CACCTT CTACAATGAGCTGCGTGTG-3’.

### Luciferase assay

LN229 cells were seeded in 24-well plates at a density of 2 × 10^4^ cells/well and transfected with plasmids using Lipofectamine 2000 transfection reagent. In each experiment, 0.5 μg of the control vector or a reporter vector (containing promoter sequences of different lengths) was co-transfected along with pRL-TK (Renilla luciferase, Promega, E2241). A dual-luciferase reporter assay system was used for measurement after the cells were exposed to prodigiosin for 48 h.

### Animal model research

LN229 cells were implanted into the right axillae of 4-week-old male athymic mice (1 × 10^7^ cells/mouse) to establish the subcutaneous xenograft model. After cell implantation, tumor growth was measured periodically with a Vernier caliper. When the length of the tumor reached 5 mm, the mice were randomly divided into two groups and treated intraperitoneally with DMSO or 5 mg/kg prodigiosin once every 3 days. After 25 days of treatment, the mice were sacrificed, and tumors were harvested for western blot analysis.

### Bioinformatic analysis

GEPIA^41^ (http://gepia.cancer-pku.cn/) was used to analyze the expression of KIAA1524 and in normal brain and tumor tissues. Glioma RNA-seq expression data were downloaded from CGGA. Univariate and multivariate proportional hazards regression models were employed to evaluate the impacts of KIAA1524. Kaplan‒Meier analysis was performed to explore the prognostic value of KIAA1524. Gene Ontology (GO) enrichment analysis of the relevant biological functions was performed. The STRING database (https://cn.string-db.org/) was used to analyze protein interactions. R language (R version 4.1.1) packages were used for other statistical computations and figures generation, and *p* < 0.05 was considered statistically significant.

### Statistical analysis

Statistical analysis was performed using unpaired Student's t test with data from at least three independent experiments. Differences with *p* < 0.05 were considered statistically significant. The statistical analyses carried out in this study were performed using R programming language (*R* version 4.1.0).

## Conclusions

In this study, we found that PG can inhibit KIAA1524 expression, thereby blocking the cell cycle by affecting the KIAA1524/PP2A/Akt signalling pathway. Our research provides strong evidence to support the value of PG in the development of antitumor drugs.

## Supplementary Information

Supplementary Information 1.

Supplementary Information 2.

Supplementary Information 3.

## Data Availability

Analysed data are presented within the current manuscript. Individual raw data is available from the corresponding author upon reasonable request.
