# Supplementary material for: Prodigiosin inhibits the proliferation of glioblastoma by regulating the KIAA1524/PP2A signaling pathway
Source: Sci Rep. 2022 Nov 2;12:18527. doi: 10.1038/s41598-022-23186-w (PMC9630538; doi:10.1038/s41598-022-23186-w)
Supplement: Supplementary file 1 — Supplementary Information 1. [file 41598_2022_23186_MOESM1_ESM.pdf]

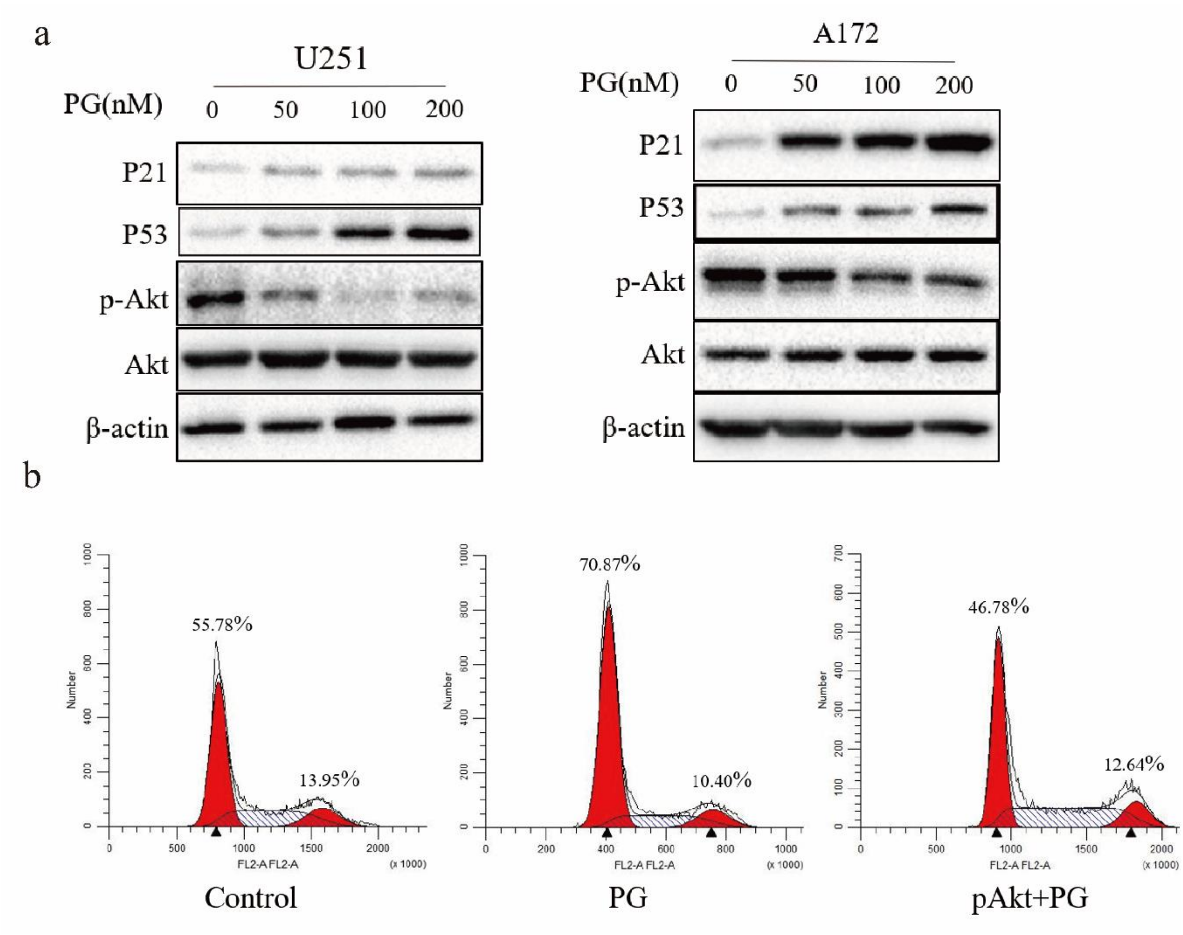

Supplemental 1(a) The protein levels of P21, P53, p-Akt(S473), Akt and β-actin in U251 and A172 cells treated with PG. Human β-actin was used as a reference gene. (b) Cell cycle analysis after expressing p-Akt or vector in LN229 cells in the presence or absence of PG (100 nM) for 48h.

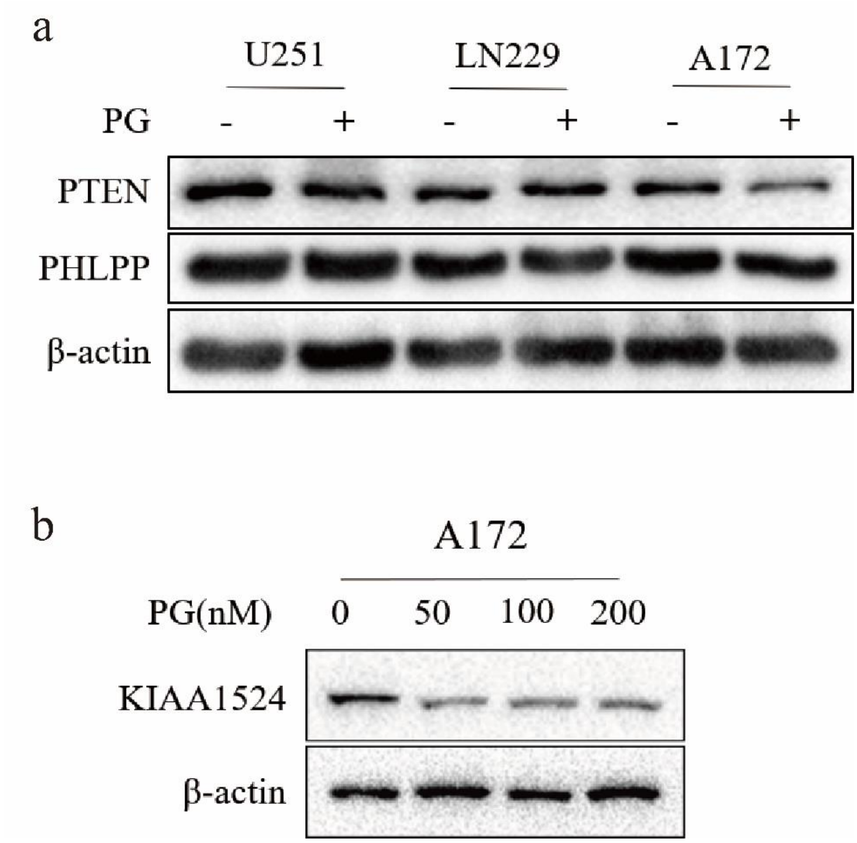

Supplemental 2(a) Western blotting analysis of PTEN and PHLPP in LN229 cells. Human β-actin was used as a reference gene. (b) Western blotting analysis of KIAA1524 expression in A172 cells treated with or without PG.
