# Supplementary material for: Prodigiosin inhibits the proliferation of glioblastoma by regulating the KIAA1524/PP2A signaling pathway
Source: Sci Rep. 2022 Nov 2;12:18527. doi: 10.1038/s41598-022-23186-w (PMC9630538; doi:10.1038/s41598-022-23186-w)
Supplement: Supplementary file 2 — Supplementary Information 2. [file 41598_2022_23186_MOESM2_ESM.pdf]

figure 2d
repetition1

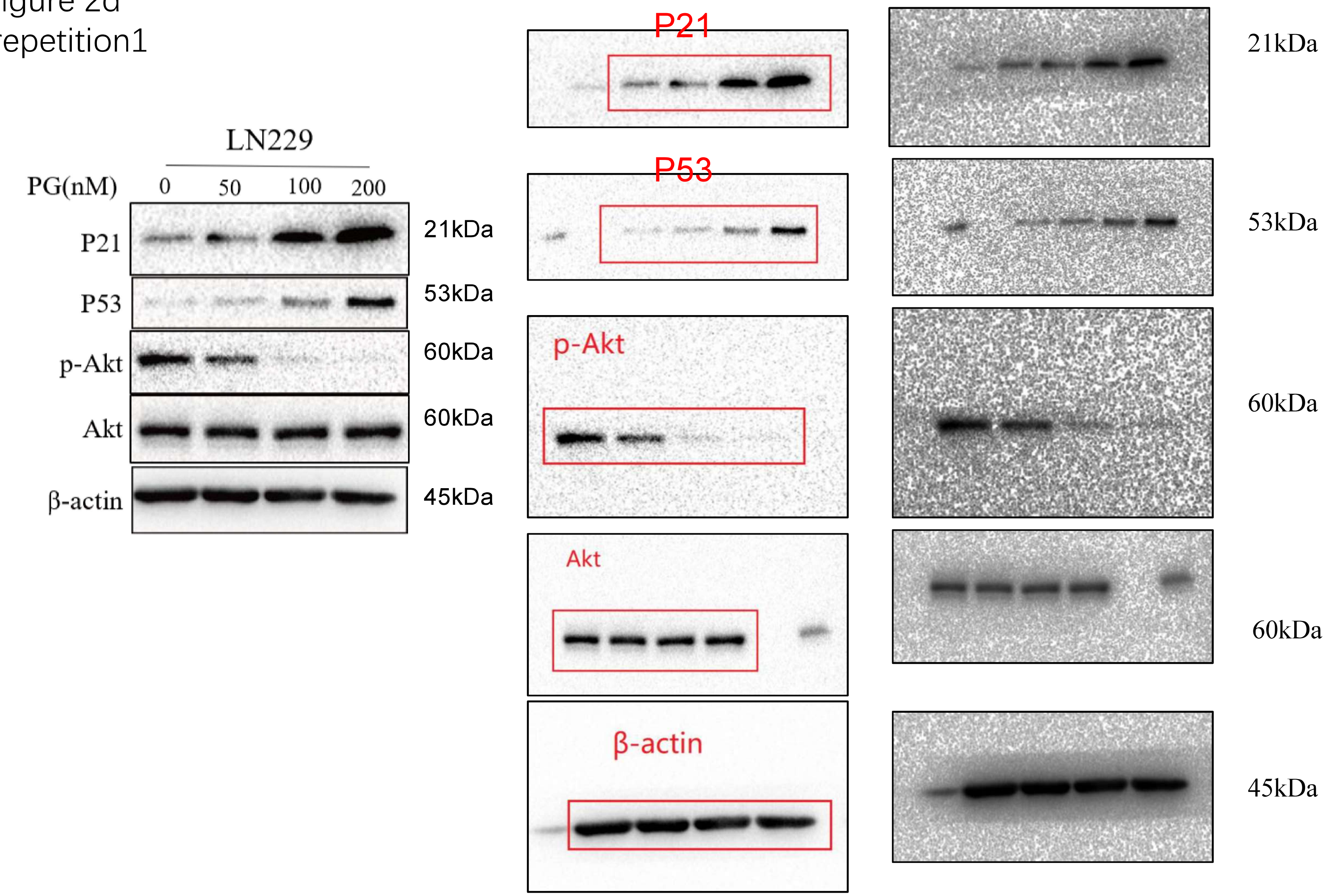

figure 2d
repetition2

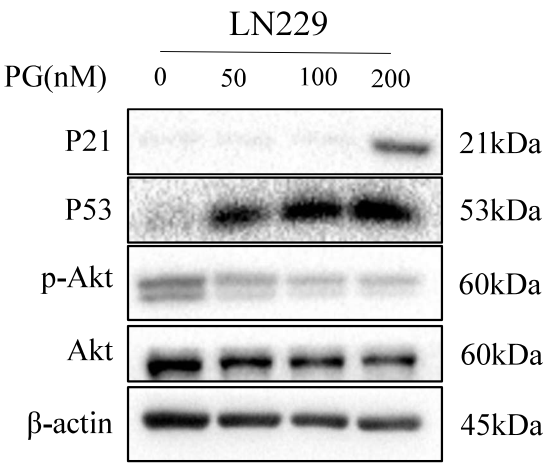

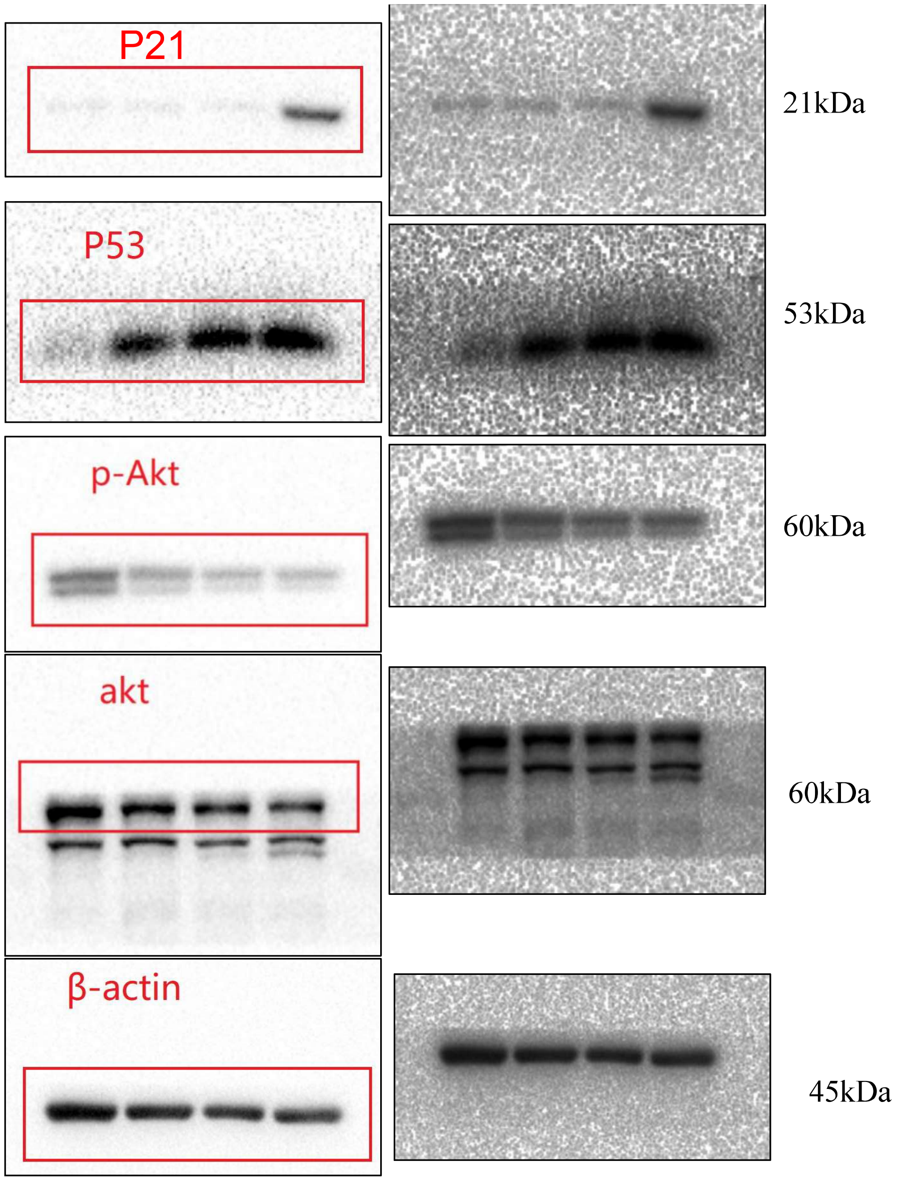

figure 2d
repetition3

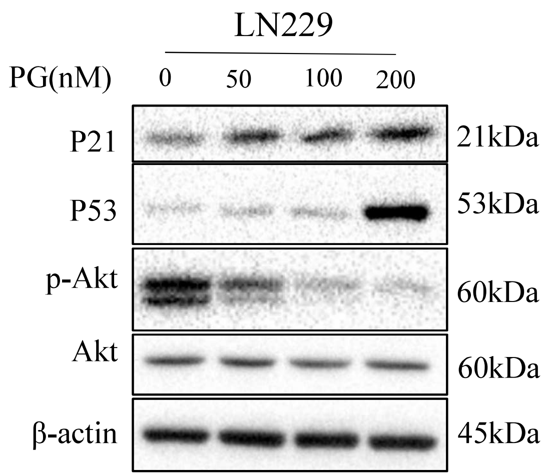

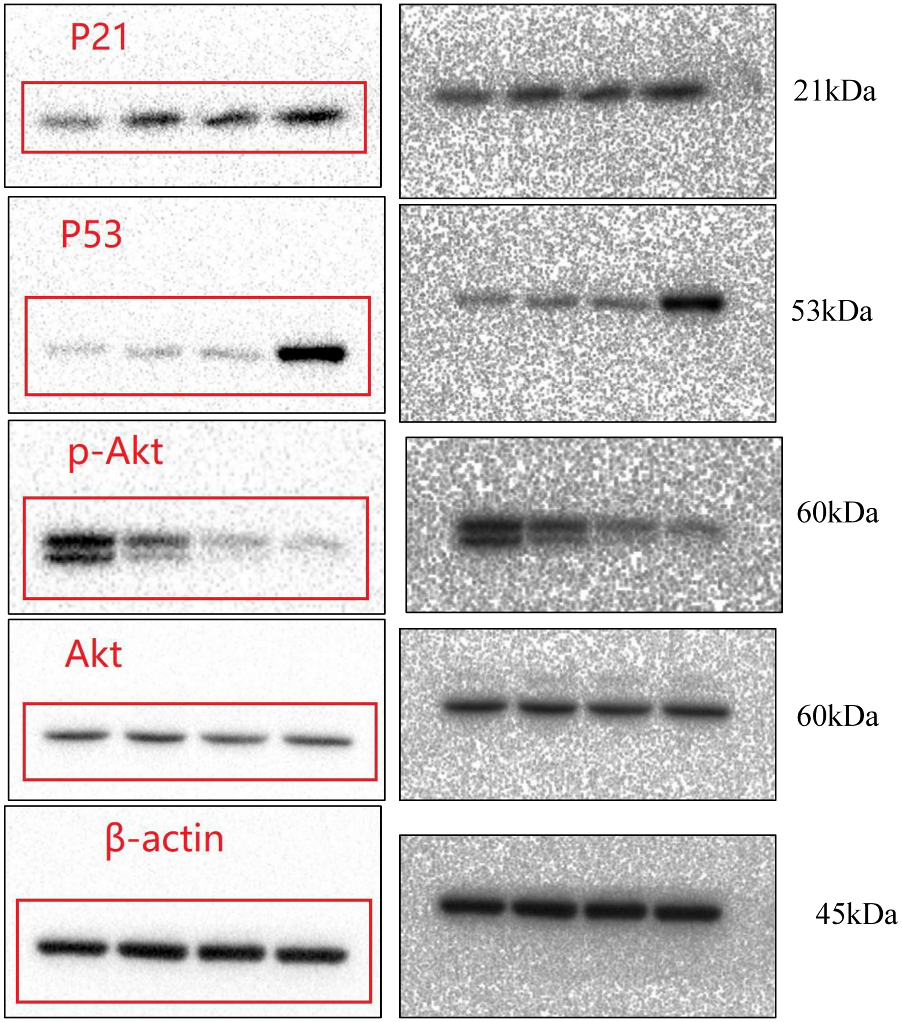

figure 2f
repetition1

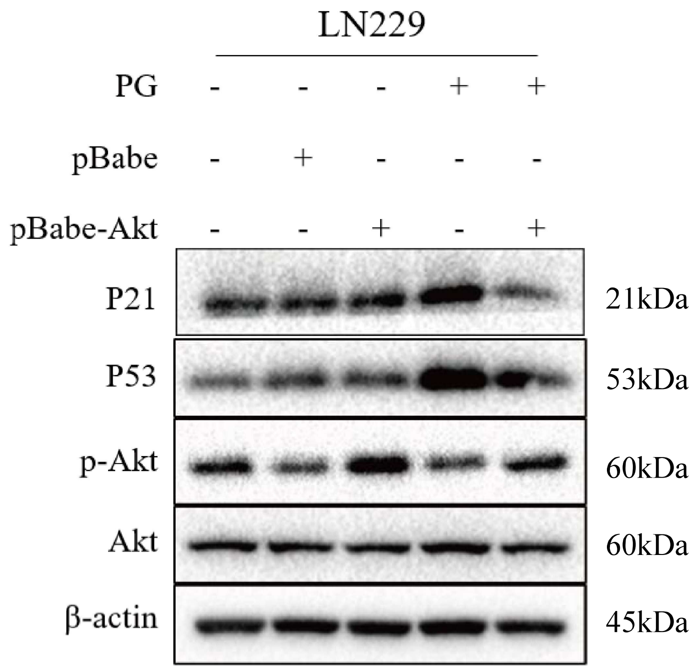

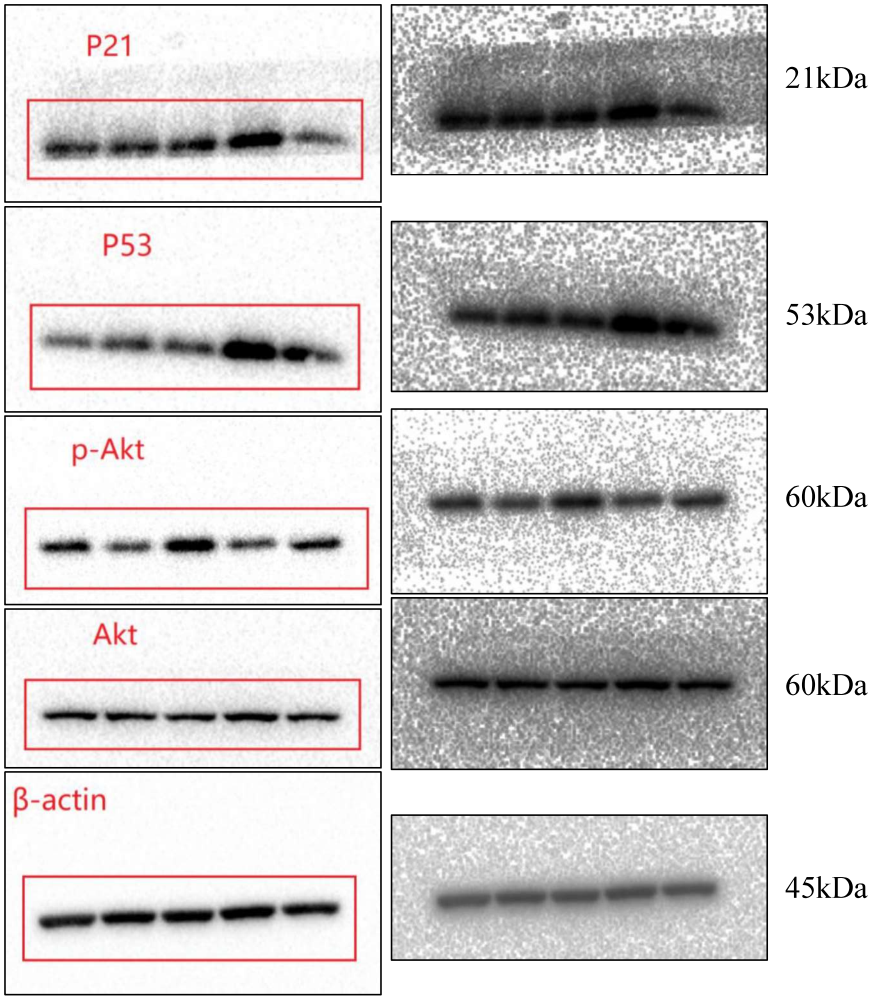

figure 2f
repetition2

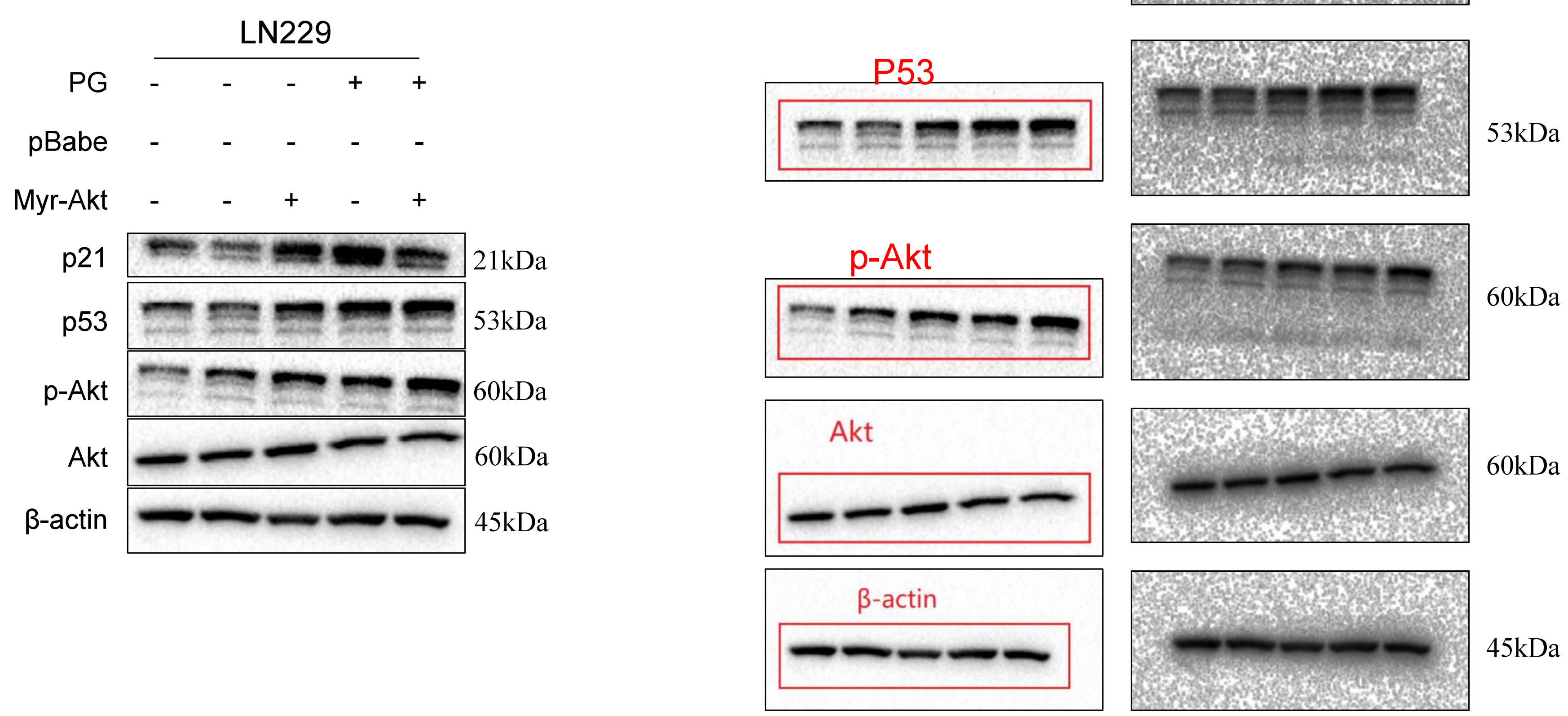

figure 2f
repetition3

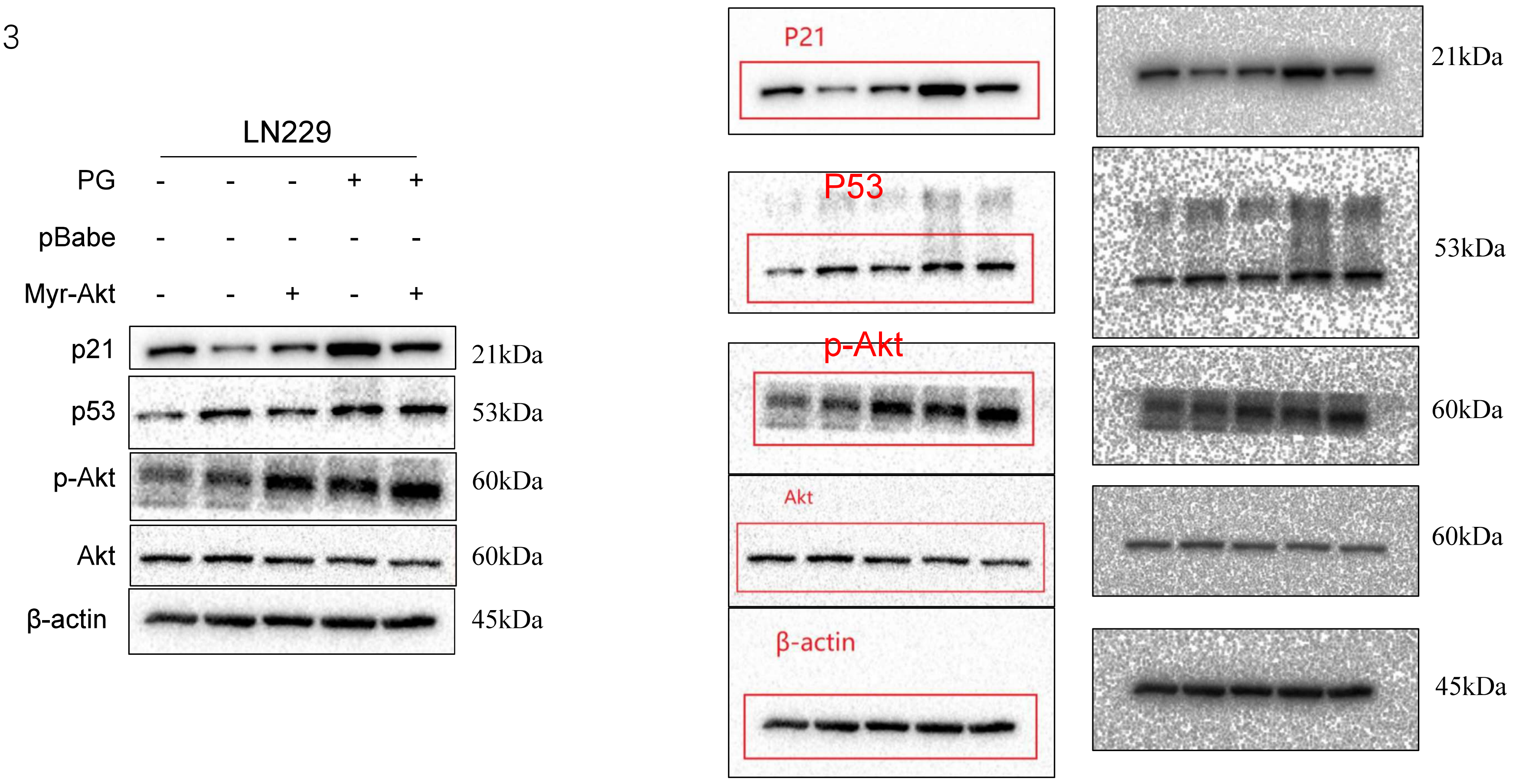

figure 3c
LN229

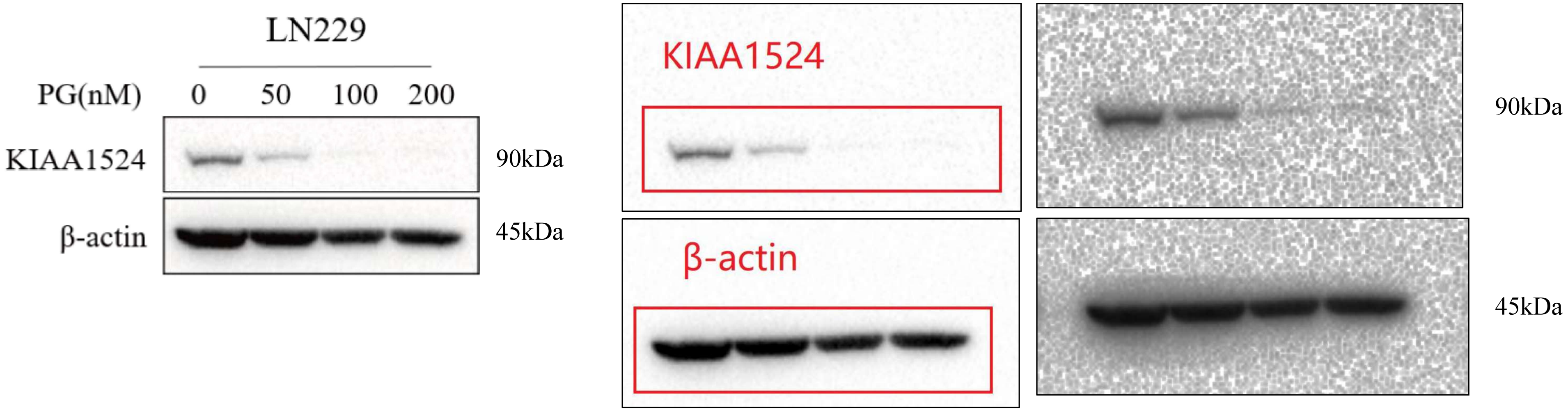

figure 3c
U251

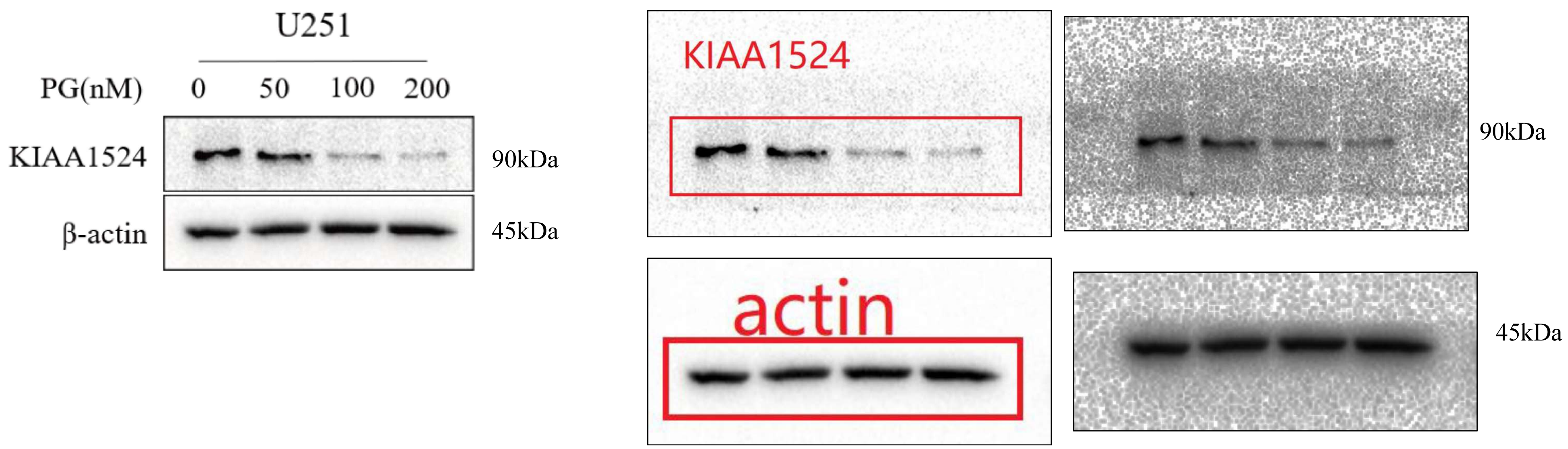

figure 3d
repetition1

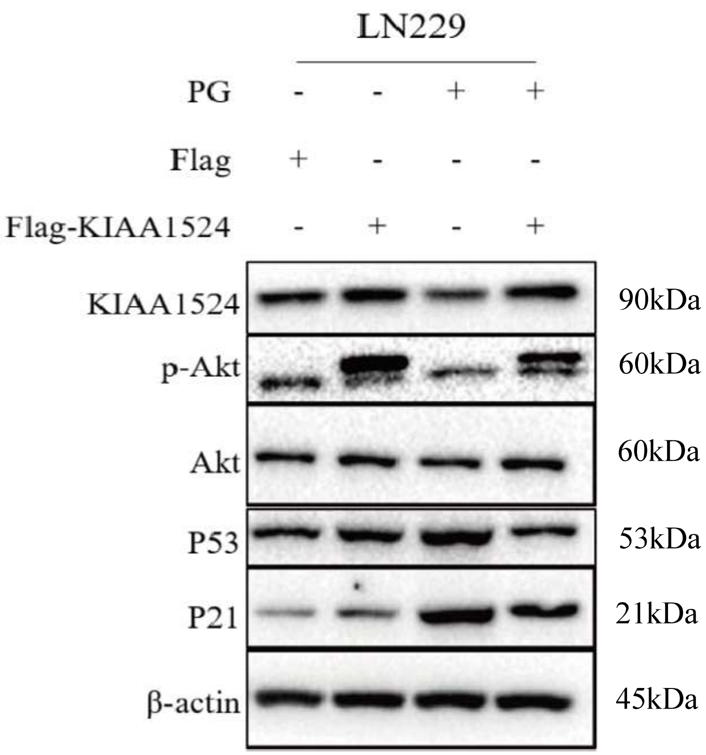

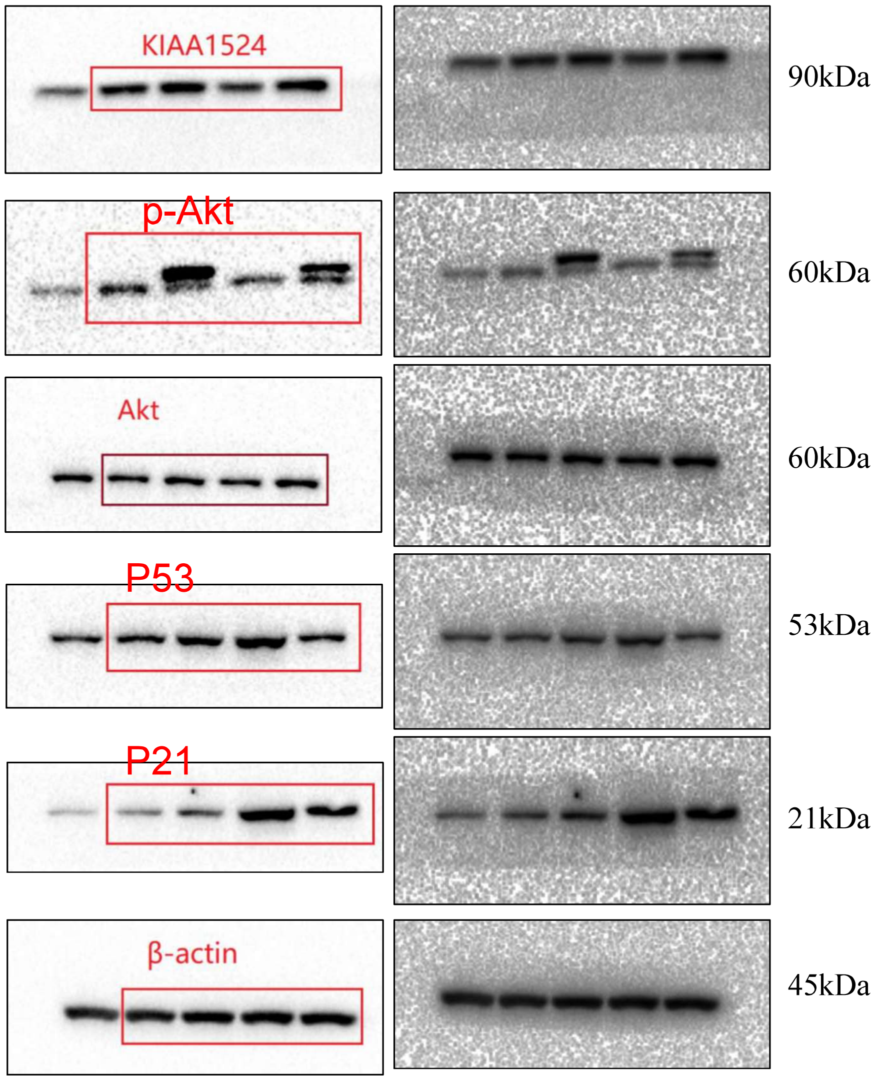

figure 3d
repetition2

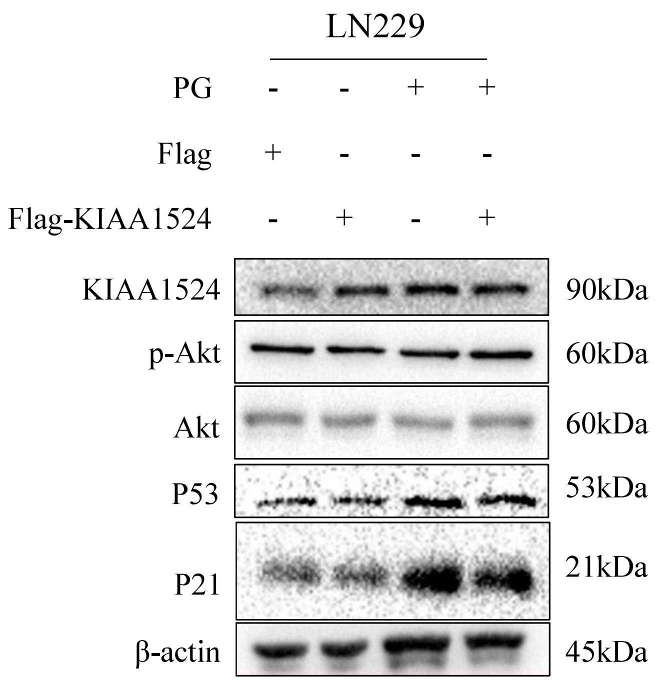

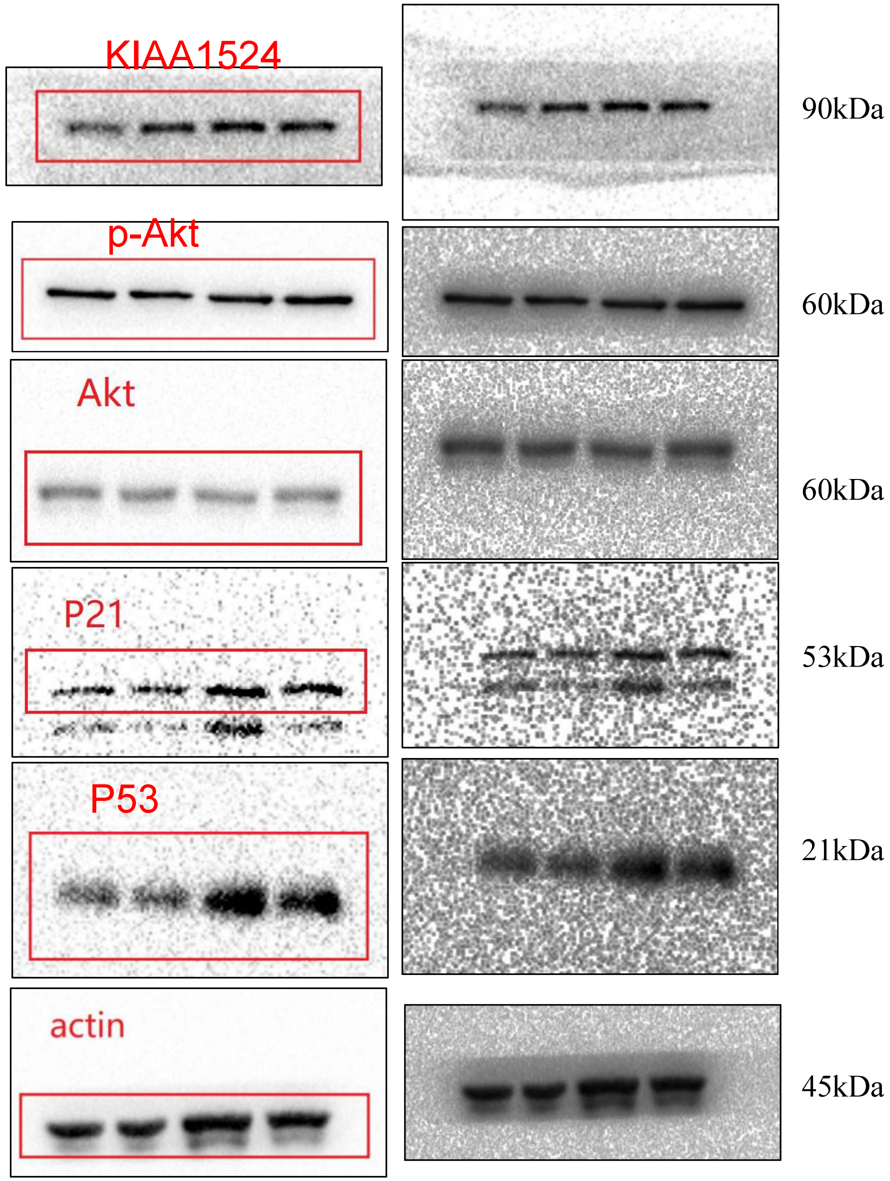

figure 3d
repetition3

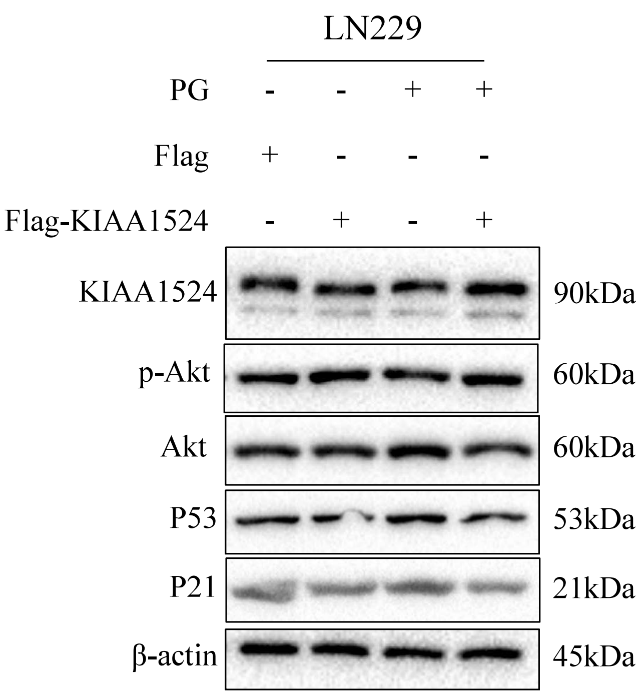

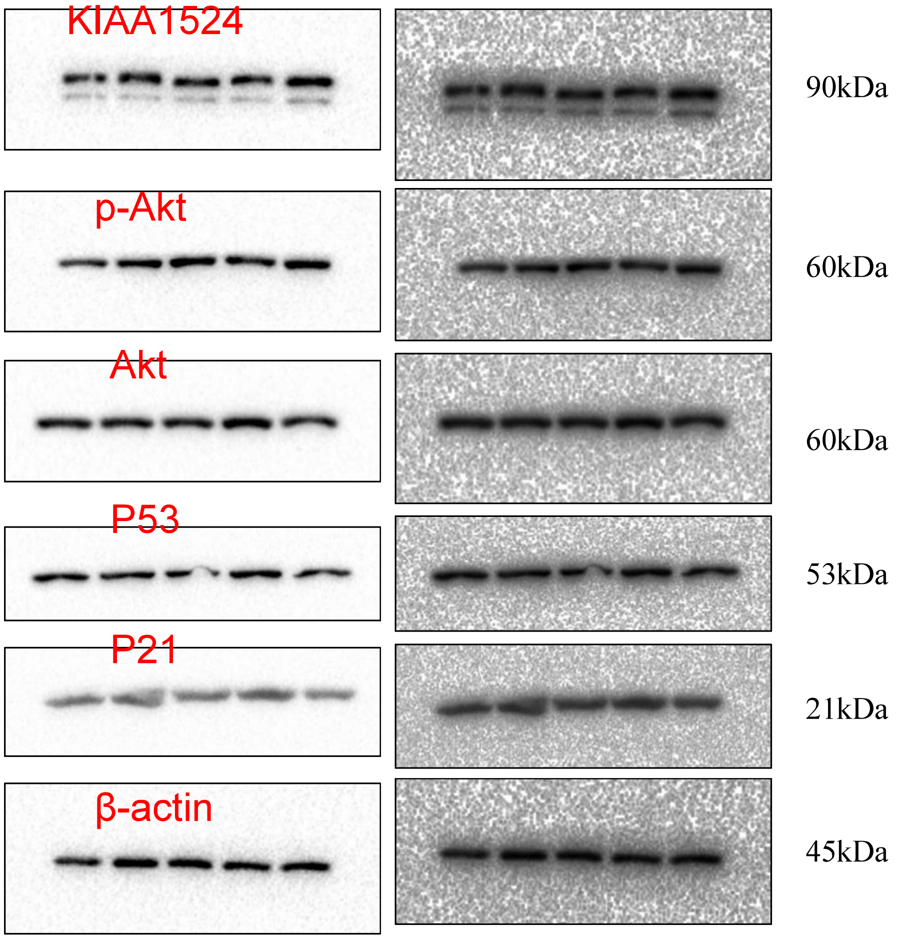

figure 5b
CHX

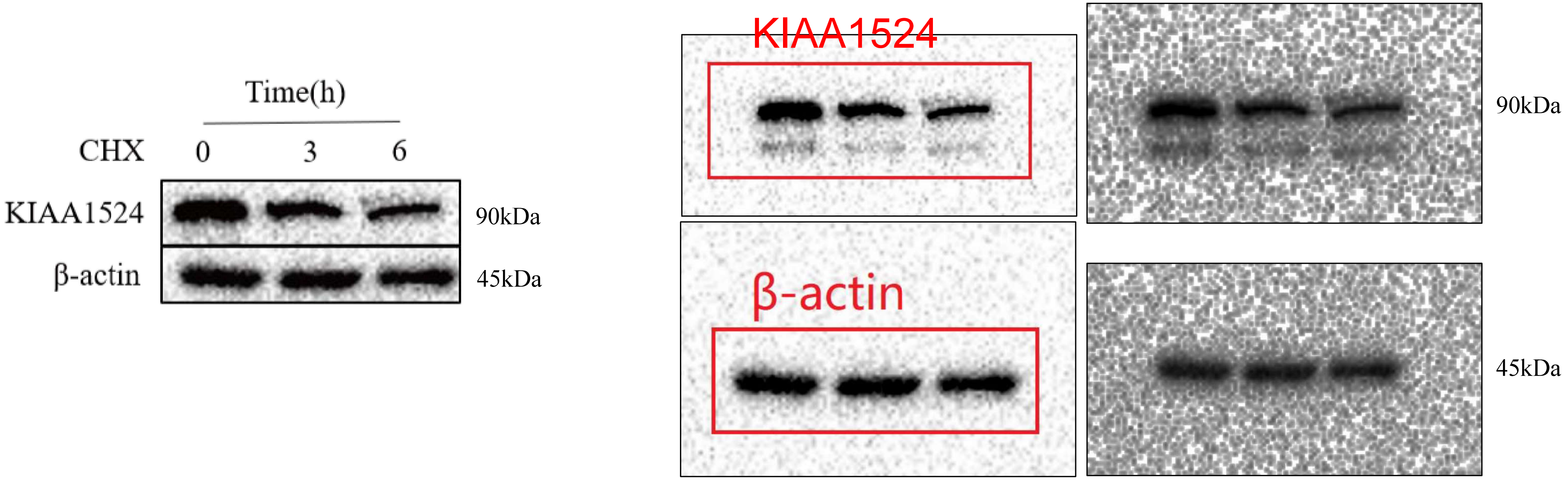

figure 5b
CHX+PG

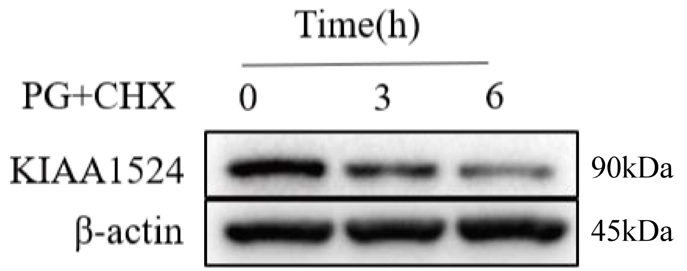

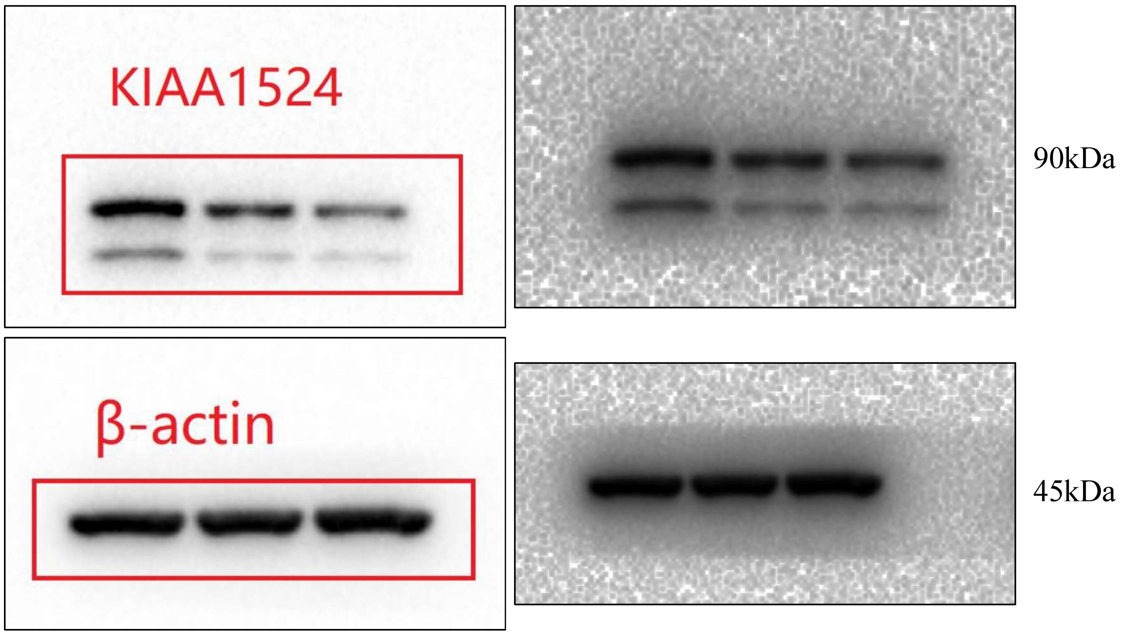

figure 5e

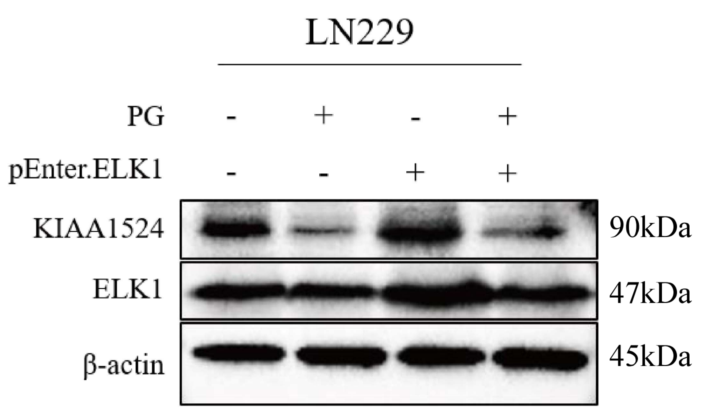
LN229
PG - + - +
pEnter.ELK1 - - + +
KIAA1524
ELK1
β-actin
90kDa
47kDa
45kDa

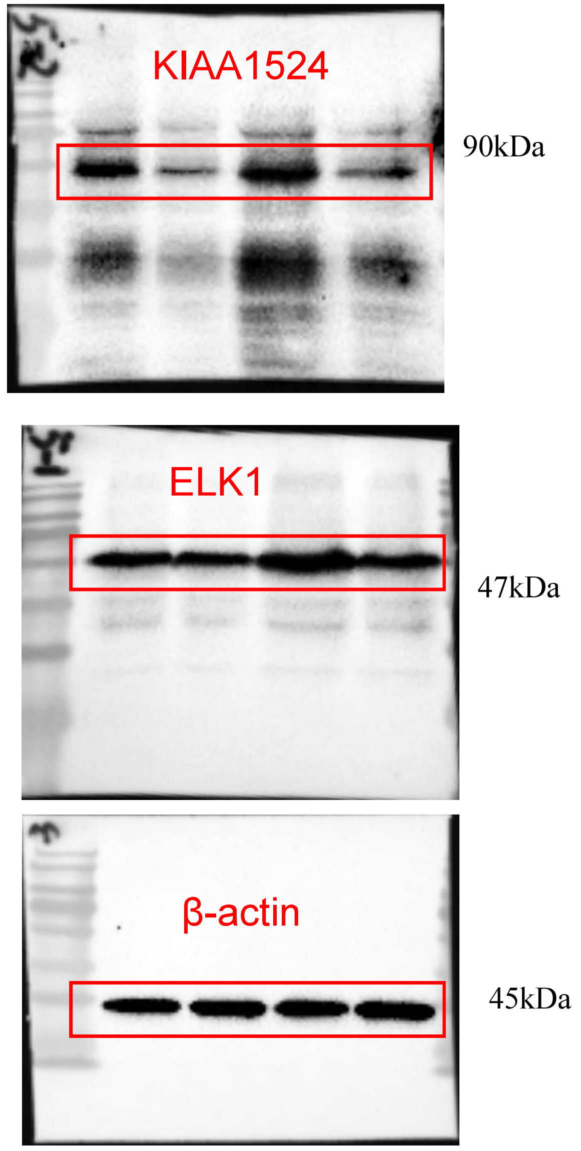
KIAA1524
90kDa
ELK1
47kDa
β-actin
45kDa

figure 6e
repetition1

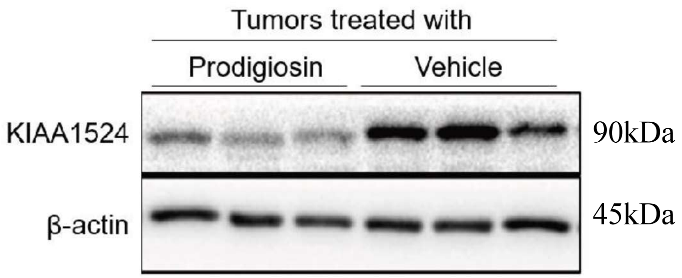

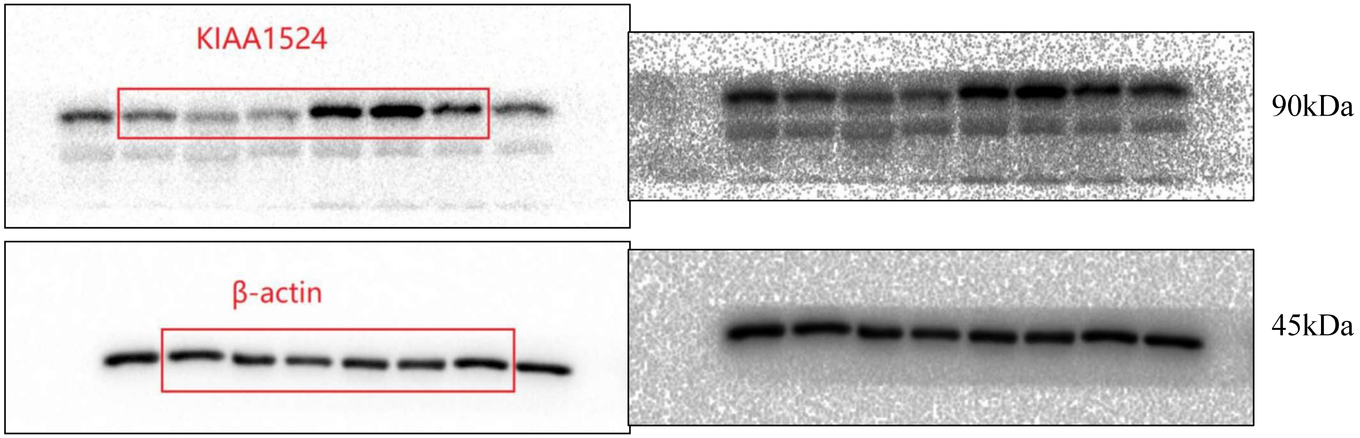

figure 6e
repetition2

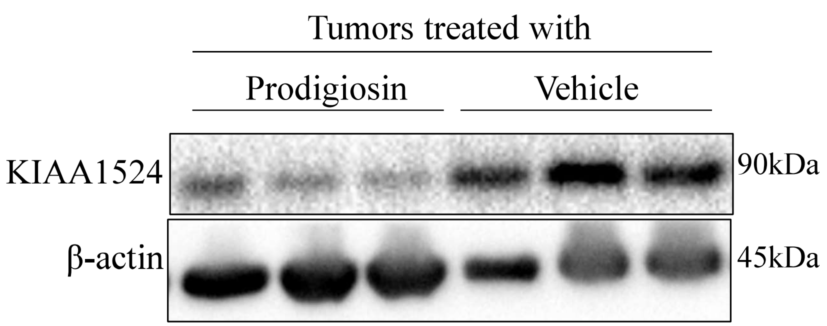

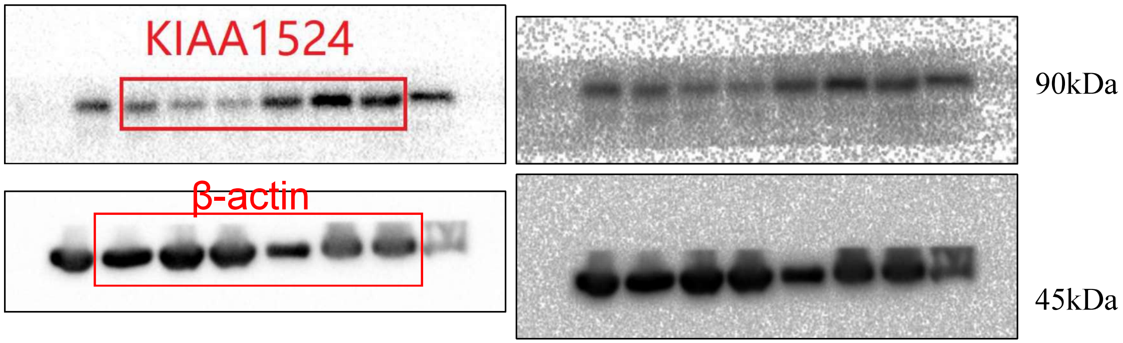

figure 6e
repetition3

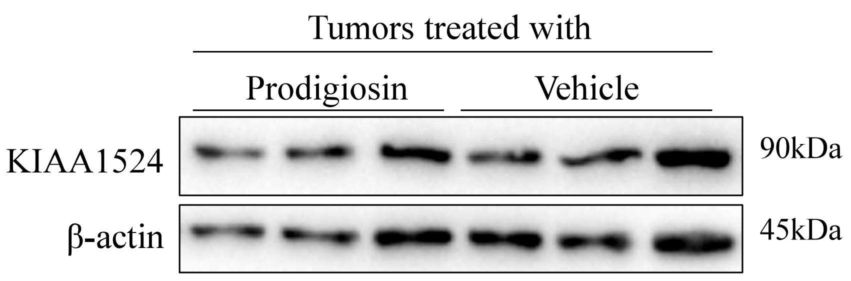

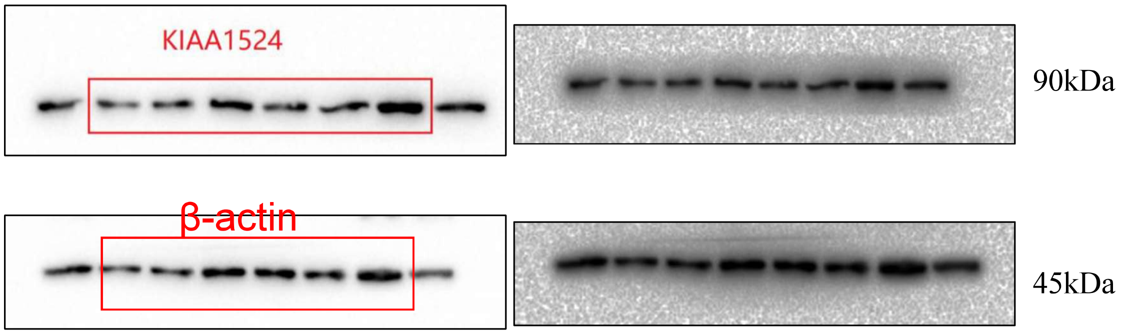

figure S1a
U251

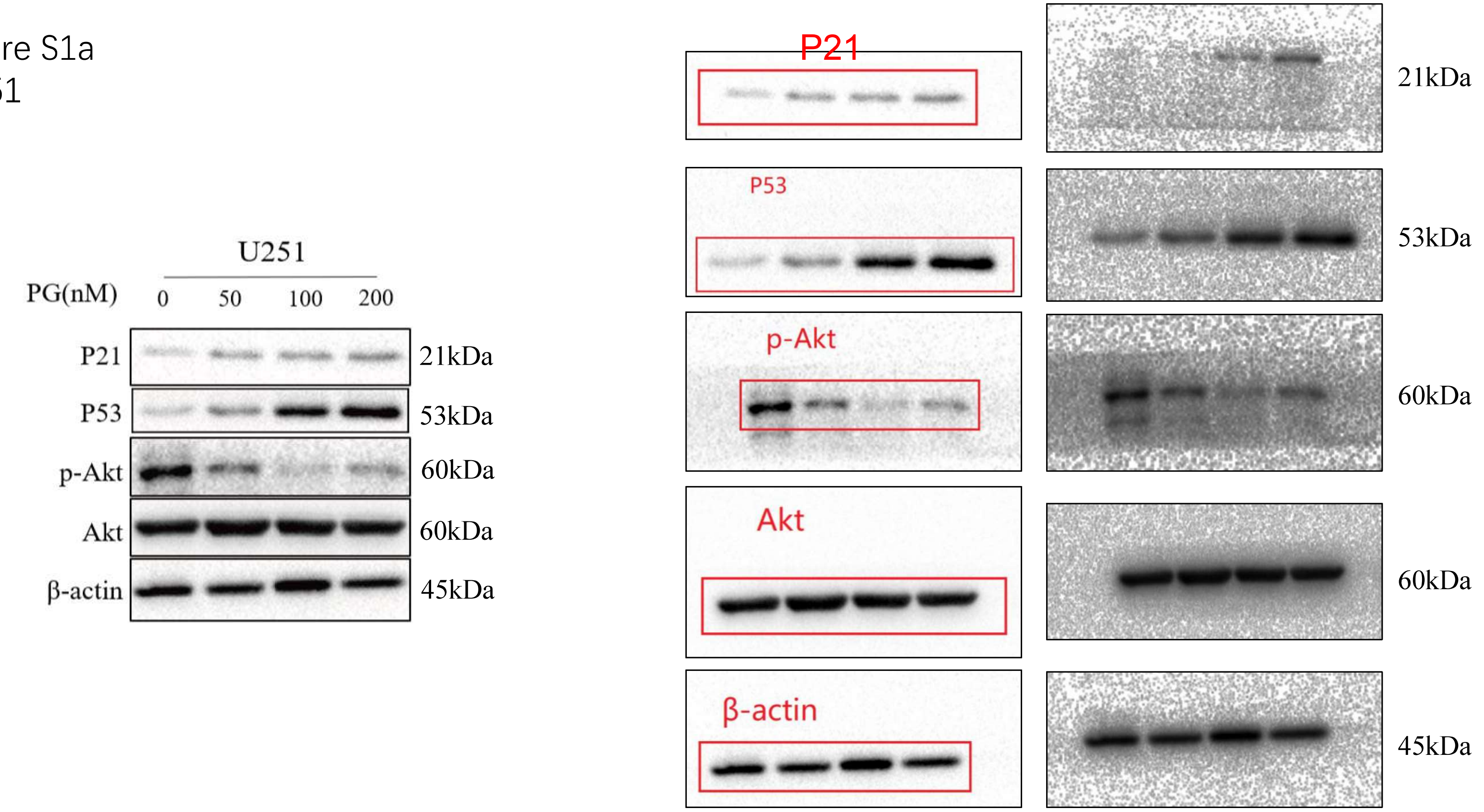

figure S1a
A172

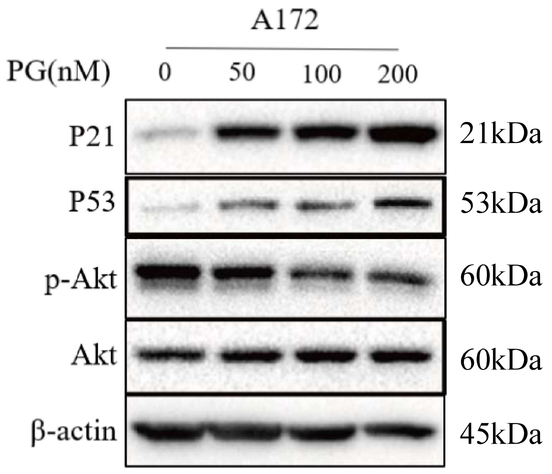

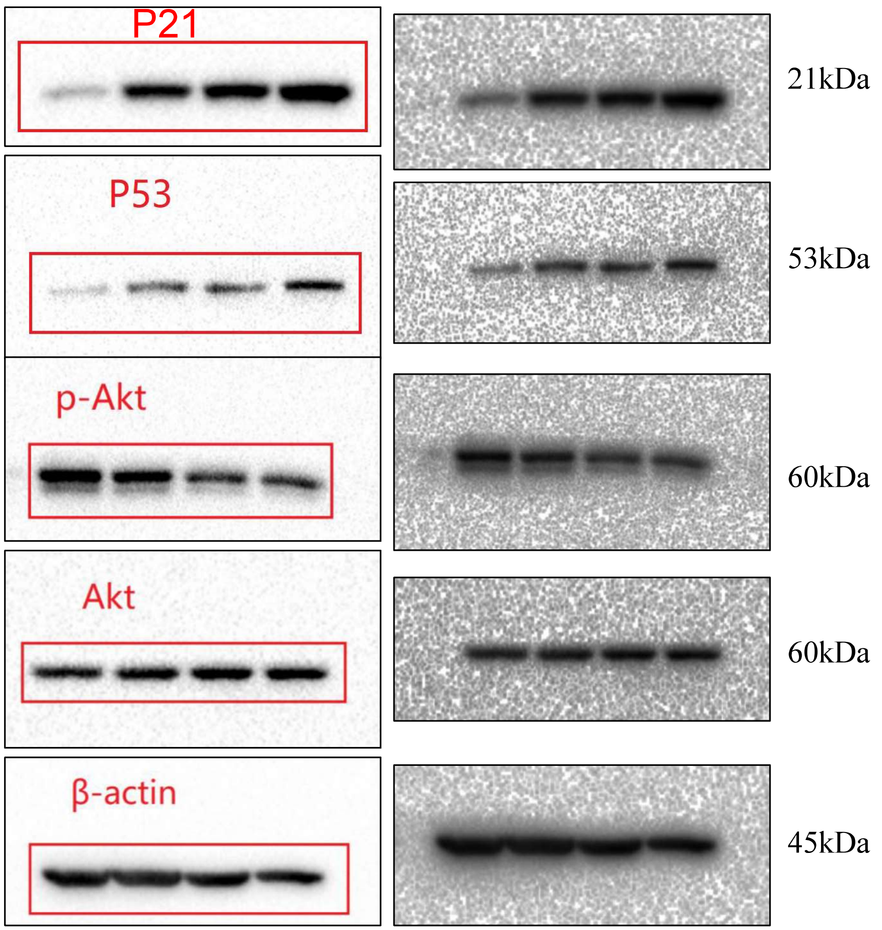

figure S2a

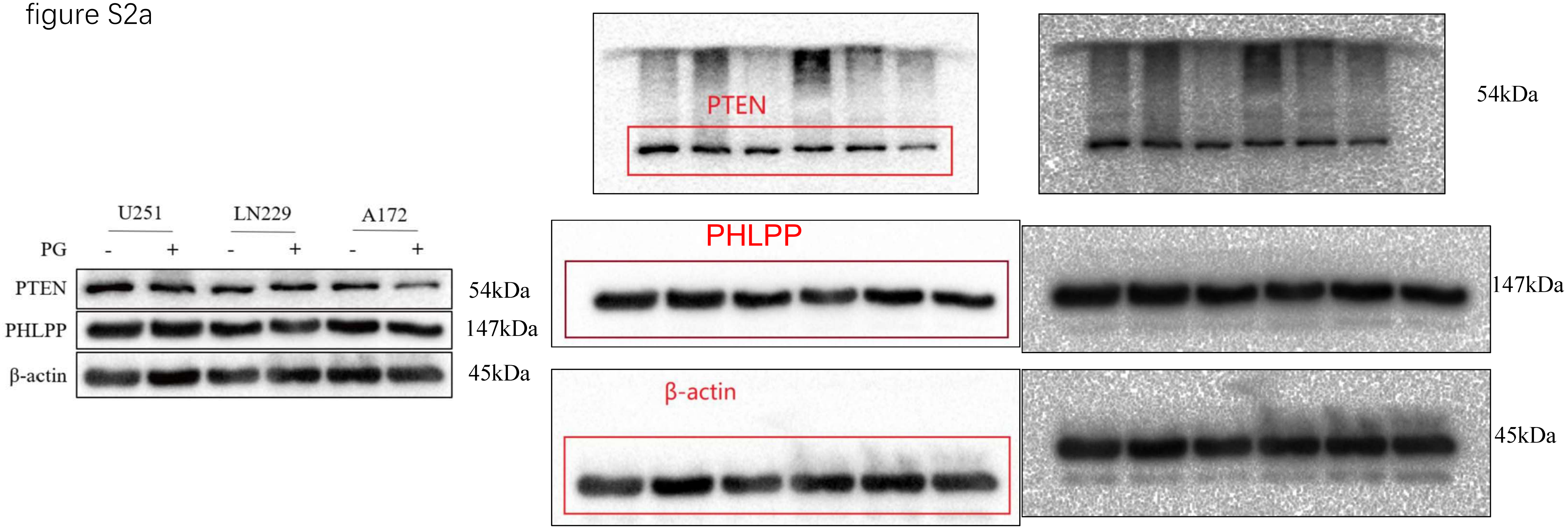
U251
LN229
A172
PG
-
+
-
+
-
+
PTEN
PHLPP
β-actin
54kDa
147kDa
45kDa
PTEN
PHLPP
β-actin
54kDa
147kDa
45kDa

figure S2b

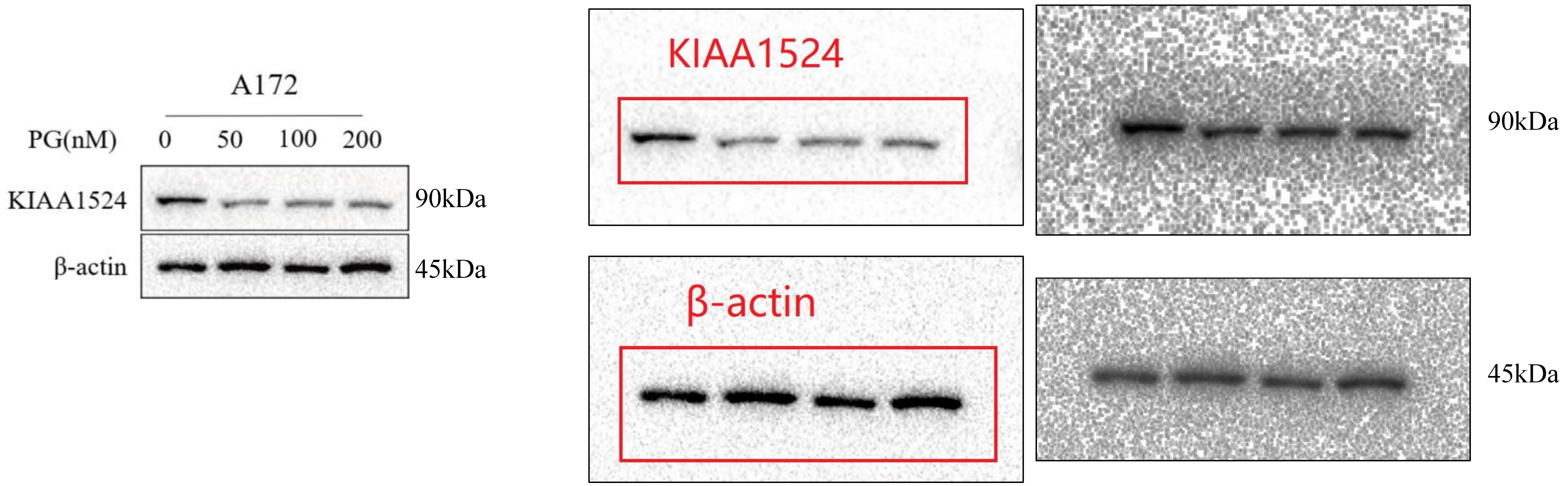

A172
PG(nM)
0
50
100
200
KIAA1524
90kDa
β-actin
45kDa
KIAA1524
β-actin
90kDa
45kDa
