# Supplementary material for: Prodigiosin inhibits the proliferation of glioblastoma by regulating the KIAA1524/PP2A signaling pathway
Source: Sci Rep. 2022 Nov 2;12:18527. doi: 10.1038/s41598-022-23186-w (PMC9630538; doi:10.1038/s41598-022-23186-w)
Supplement: Supplementary file 3 — Supplementary Information 3. [file 41598_2022_23186_MOESM3_ESM.doc]

| B5854-1 | AGGGTTCCAAGCTTAAGCGGCCGCGCCACCATGGACTCCACTGCCTGCTTGAAGTCCTTGCTCC |
| --- | --- |
| B5854-2 | GTTCGCCTCTGACTTCACGGCTTTGTACTGACTGACAGTCAGGAGCAAGGACTTCAAGCAGGCAG |
| B5854-3 | CAAAGCCGTGAAGTCAGAGGCGAACGCCACTCAGCTTTTGCGGCACTTGGAGGTAATTTCTGGAC |
| B5854-4 | GTTAATATCTGATTTGATGTAAATAGTCGTGTGAGTTTCTGTCCAGAAATTACCTCCAAGTGCCG |
| B5854-5 | TATTTACATCAAATCAGATATTAACAAGTGAATGCTTGAGTTGCCTTGTAGAGCTACTTGAAGAC |
| B5854-6 | AACCGATAATACTTAAGATCAGTGAAGCACTTATGTTGGGGTCTTCAAGTAGCTCTACAAGGCAA |
| B5854-7 | TCACTGATCTTAAGTATTATCGGTTTGCTGTCTCAACTAGCAGTAGACATTGAAACCAGAGATTG |
| B5854-8 | TCCCGCCAGCACACTATTCAGATTATATGTATTCTGAAGACAATCTCTGGTTTCAATGTCTACTG |
| B5854-9 | TAATCTGAATAGTGTGCTGGCGGGAGTGGTTTGTCGGAGCAGCCACACTGATTCGGTGTTTTTGC |
| B5854-10 | TTGACATTATATGTTAACTTCTGTAGAAGTTGAATGCACTGCAAAAACACCGAATCAGTGTGGCT |
| B5854-11 | TACAGAAGTTAACATATAATGTCAAAATTTTCTATTCTGGTGCCAATATAGATGAATTAATTACG |
| B5854-12 | TTAACTCATCTTCAGAAGATTGAATGTGATCTATCAGGAACGTAATTAATTCATCTATATTGGCA |
| B5854-13 | ATTCAATCTTCTGAAGATGAGTTAAAAATGCCTTGTCTAGGATTATTGGCAAATCTTTGTCGGCA |
| B5854-14 | ATTACTCAATGTCTTTATGTGCGTTTGAACAGAAAGATTGTGCCGACAAAGATTTGCCAATAATC |
| B5854-15 | AACGCACATAAAGACATTGAGTAATGTGAAATCTTTTTATCGAACTCTTATCACCTTGTTGGCCC |
| B5854-16 | AATATTGAAAGTGCAAACACAACCACAGTTAAACTACTATGGGCCAACAAGGTGATAAGAGTTCG |
| B5854-17 | TGGTTGTGTTTGCACTTTCAATATTATCCAGTTTGACATTAAATGAAGAGGTGGGGGAAAAGCTA |
| B5854-18 | ATGTTTCGAGCATGGAATAGCTTTTCCCCCACCTCTTCATTT |
| B5854-19 | ATGAAGAGGTGGGGGAAAAGCTATTCCATGCTCGAAACATTCATCAGACTTTTCAACTAATATTT |
| B5854-20 | ACTTTCTAGTTAGAGTGCCATCACCGTTTATGAGAATATTAAATATTAGTTGAAAAGTCTGATGA |
| B5854-21 | GGTGATGGCACTCTAACTAGAAAGTATTCAGTTGACCTACTGATGGATCTCCTTAAGAATCCTAA |
| B5854-22 | TGAAGAAAAGTGCTCATATCTGGTGAGATAATCAGCAATTTTAGGATTCTTAAGGAGATCCATCA |
| B5854-23 | CACCAGATATGAGCACTTTTCTTCATGTCTTCACCAAGTATTAGGTCTTCTTAATGGAAAGGATC |
| B5854-24 | AAGGCAAGAAGTAATTCTAAAACCTTTGAAGAGGAATCAGGATCCTTTCCATTAAGAAGACCTAA |
| B5854-25 | AGGTTTTAGAATTACTTCTTGCCTTCTGTTCAGTGACTCAGCTGCGCCATATGCTCACTCAGATG |
| B5854-26 | CAGAGTGGCGCTGCCAGGTGGAGACTGTTCAAACATCATCTGAGTGAGCATATGGCGCAGC |
| B5854-27 | CCACCTGGCAGCGCCACTCTGGGAAGCCATACTAAATGTTTAGAACCTACTGTGGCTCTACTGCG |
| B5854-28 | AGAACAGTTTTCTGATCCGTCCAAAGGTTGGCTTAACCAGCGCAGTAGAGCCACAGTAGGTTCTA |
| B5854-29 | TTTGGACGGATCAGAAAACTGTTCTGTTTTAGCATTGGAGTTGTTCAAGGAAATATTTGAGGATG |
| B5854-30 | ACAAAACGATCAGCCGAGGAACAGTTAGCAGCATCTATGACATCCTCAAATATTTCCTTGAACAA |
| B5854-31 | ACTGTTCCTCGGCTGATCGTTTTGTGACCCTTCTGCTGCCTACAATCCTTGATCAACTTCAGTTC |
| B5854-32 | ATTTTTTTCTTGTTAAAGCCTCATCTAGATTTTGTTCTGTGAACTGAAGTTGATCAAGGATTGTA |
| B5854-33 | GATGAGGCTTTAACAAGAAAAAAATGTGAAAGGATTGCCAAGGCCATTGAAGTTTTGTTAACTCT |
| B5854-34 | GATTTTTGCAATATGCATTTTTAGTGTATCATCTCCACAGAGAGTTAACAAAACTTCAATGGCCT |
| B5854-35 | ACTAAAAATGCATATTGCAAAAATCTTGACAACTGTCAAGTGTACCACTCTTATAGAACAACAAT |
| B5854-36 | AATCTTGCCATATGTAAATTGTTGTTCTATAAGAGTGGTACA |
| B5854-37 | TACCACTCTTATAGAACAACAATTTACATATGGCAAGATTGACCTGGGATTTGGAACAAAGGTTG |
| B5854-38 | AAATTACATCAGCAGCAAGTTTGCATAATTCAGAATCTGCAACCTTTGTTCCAAATCCCAGGTCA |
| B5854-39 | ATGCAAACTTGCTGCTGATGTAATTTTGAAAACTCTTGATTTGATTAACAAACTTAAACCATTGG |
| B5854-40 | CCTGAAGTATTTTGTAGAAGCTTACTTCCATACCAGGAACCAATGGTTTAAGTTTGTTAATCAAA |
| B5854-41 | AGTAAGCTTCTACAAAATACTTCAGGACCCACGTTTGATTACTCCTTTGGCTTTTGCTTTAACGT |
| B5854-42 | ATATTCTCAGTCCAGACTGTACTTGTTCTCTATTATCTGACGTTAAAGCAAAAGCCAAAGGAGTA |
| B5854-43 | ACAAGTACAGTCTGGACTGAGAATATTATTGGAGGCTGCTCCACTGCCAGATTTTCCTGCTTTAG |
| B5854-44 | GTCTATAGGCATTGTTTGCTGCTATACTTTCTCCAAGTACTAAAGCAGGAAAATCTGGCAGTGGA |
| B5854-45 | TATAGCAGCAAACAATGCCTATAGACAACAGGAAACAGAACATATACCCAGAAAAATGCCCTGGC |
| B5854-46 | AACACTTTATTGATGTTGGAAAACTGTGATTTGATGATTGCCAGGGCATTTTTCTGGGTATATGT |
| B5854-47 | CAGTTTTCCAACATCAATAAAGTGTTTAACTCCTCATTTGAAAGATGGTGTTCCTGGATTGAATA |
| B5854-48 | CCACCATTCCAGACTGAAGTTTCTCTATTAATTCTTCAATATTCAATCCAGGAACACCATCTTTC |
| B5854-49 | AGAGAAACTTCAGTCTGGAATGGTGGTAAAGGATCAGATTTGTGATGTGAGAATATCTGACATAA |
| B5854-50 | TGGAAGCTAATGTGGATAGTTTCATTTCATATACATCCATTATGTCAGATATTCTCACATCACAA |
| B5854-51 | AATGAAACTATCCACATTAGCTTCCAAAGAAAGCAGGCTACAAGATCTTTTGGAAACAAAAGCTC |
| B5854-52 | GATGCTGAGCAATCAGTCTATCAGCCTGTGCAAGGGCTAGAGCTTTTGTTTCCAAAAGATCTTGT |
| B5854-53 | GGCTGATAGACTGATTGCTCAGCATCGCTGTCAAAGAACTCAAGCTGAAACAGAGGCACGGACAC |
| B5854-54 | TTTTTTCTCTCAACTTCTCTCAACATACTAGCAAGTGTCCGTGCCTCTGTTTCAGCTTGA |
| B5854-55 | GGACACTTGCTAGTATGTTGAGAGAAGTTGAGAGAAAAAATGAAGAGCTTAGTGTGTTGCTGAAG |
| B5854-56 | AATATCACTCTGCGCTCTTTCTGATTCAACTTGCTGCGCCTTCAGCAACACACTAAGCTCTTCAT |
| B5854-57 | AATCAGAAAGAGCGCAGAGTGATATTGAGCATCTCTTTCAACATAATAGGAAGTTAGAGTCTGTG |
| B5854-58 | TTCCATGTAGGATTTTGTCAGTATTTCATGTTCTTCAGCCACAGACTCTAACTTCCTATTATGTT |
| B5854-59 | AAATACTGACAAAATCCTACATGGAACTTCTTCAGAGAAATGAAAGTACTGAAAAGAAGAATAAA |
| B5854-60 | AATTTGTTTATTCAGAGAATCACATGTGATCTGTAAATCTTTATTCTTCTTTTCAGTACTTTCAT |
| B5854-61 | CATGTGATTCTCTGAATAAACAAATTGAGACAGTGAAAAAGTTGAATGAGTCACTCAAGGAACAA |
| B5854-62 | TTCTTCTTTCTCTATTAATTGGGCAATACTTTTTTCATTTTGTTCCTTGAGTGACTCATTCAACT |
| B5854-63 | TTGCCCAATTAATAGAGAAAGAAGAACAGAGAAAAGAAGTACAGAATCAGCTAGTAGACAGAGAA |
| B5854-64 | TTGTACTTTTGTTTTTTGATGCAAATTTGCTAGCTTATGTTCTCTGTCTACTAGCTGATTCTGTA |
| B5854-65 | ATTTGCATCAAAAAACAAAAGTACAAGAAGAAAAGATTAAAACCTTACAAAAGGAAAGGGAAGAT |
| B5854-66 | GCTTAATTCTTTTCTAAGGATATCAATGGTTTCTTCCTTATCTTCCCTTTCCTTTTGTAAGGTTT |
| B5854-67 | TTGATATCCTTAGAAAAGAATTAAGCAGAACAGAACAGATAAGAAAAGAGTTGAGCATTAAGGCT |
| B5854-68 | ACGACCTTCTAATTGTGCCTTTTGAACCTCTAGGGAGGAAGCCTTAATGCTCAACTCTTTTCTTA |
| B5854-69 | TTCAAAAGGCACAATTAGAAGGTCGTTTGGAAGAGAAAGAGTCCTTGGTGAAACTTCAGCAAGAG |
| B5854-70 | GTGGATCATTGCTATCATGTGGGAGTGTTTGTTCAATTCCTCTTGCTGAAGTTTCACCAAGGACT |
| B5854-71 | ACTCCCACATGATAGCAATGATCCACAGTTTAAGTGGTGGAAAAATAAATCCAGAAACTGTGAAT |
| B5854-72 | ATCAGTAGAGAGTGTCGGATCCCTATATACTGAGATTCACAGTTTCTGGATTTATTTTTC |
